# Secondary structures in RNA synthesis, splicing and translation

**DOI:** 10.1016/j.csbj.2022.05.041

**Published:** 2022-05-27

**Authors:** Ilias Georgakopoulos-Soares, Guillermo E. Parada, Martin Hemberg

**Affiliations:** a Department of Bioengineering and Therapeutic Sciences, University of California San Francisco, San Francisco, CA, USA; b Institute for Human Genetics, University of California San Francisco, San Francisco, CA, USA; c Donnelly Centre for Cellular and Biomolecular Research, University of Toronto, Toronto, ON M5S 3E1, Canada; d Department of Molecular Genetics, University of Toronto, Toronto, ON M5A 1A8, Canada; e Evergrande Center for Immunologic Diseases, Harvard Medical School and Brigham and Women’s Hospital, Boston, MA, USA

## Abstract

Even though the functional role of mRNA molecules is primarily decided by the nucleotide sequence, several properties are determined by secondary structure conformations. Examples of secondary structures include long range interactions, hairpins, R-loops and G-quadruplexes and they are formed through interactions of non-adjacent nucleotides. Here, we discuss advances in our understanding of how secondary structures can impact RNA synthesis, splicing, translation and mRNA half-life. During RNA synthesis, secondary structures determine RNA polymerase II (RNAPII) speed, thereby influencing splicing. Splicing is also determined by RNA binding proteins and their binding rates are modulated by secondary structures. For the initiation of translation, secondary structures can control the choice of translation start site. Here, we highlight the mechanisms by which secondary structures modulate these processes, discuss advances in technologies to detect and study them systematically, and consider the roles of RNA secondary structures in disease.

## Graphical abstract

**Figure:**
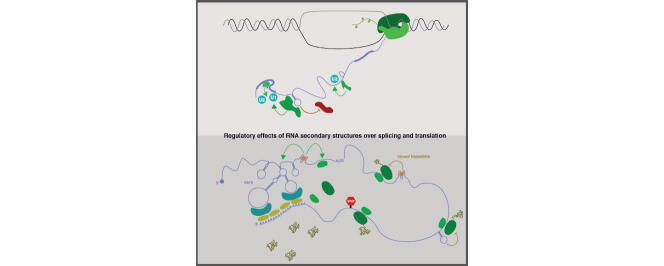

## 1 Introduction

mRNAs are essential molecules in the cell as they are key to extracting information stored in the DNA. Although the function of mRNA molecules is primarily determined by the nucleotide sequence, some properties are determined by secondary structures. Secondary structures are defined as distinct features, including hairpins, long range interactions, G-quadruplexes, R-loops and pseudoknots and they are formed as a consequence of the interactions of non-adjacent nucleotides. Their presence can impact various processes involving the mRNA, including synthesis, splicing and translation. Secondary structures are dynamic and can be modulated by multiple proteins, in particular RNA binding proteins (RBPs), and as they cannot be predicted solely from the primary sequence they are challenging to study. Nevertheless, several assays are available for both *in vitro* and *in vivo* profiling, and in this Review, we summarize these methods, provide an overview of some of the elucidated and putative functional roles of mRNA secondary structures, and finally we discuss their impact on disease. We discuss the consequences of secondary structure formation for splicing and translation, with particular focus in G-quadruplexes, hairpins and long range interactions. We also discuss the contribution of secondary structures in the regulation of mRNA splicing and in translation initiation and discuss the mechanisms involved.

## 2 RNA secondary structure formation

**Fig. 1:**
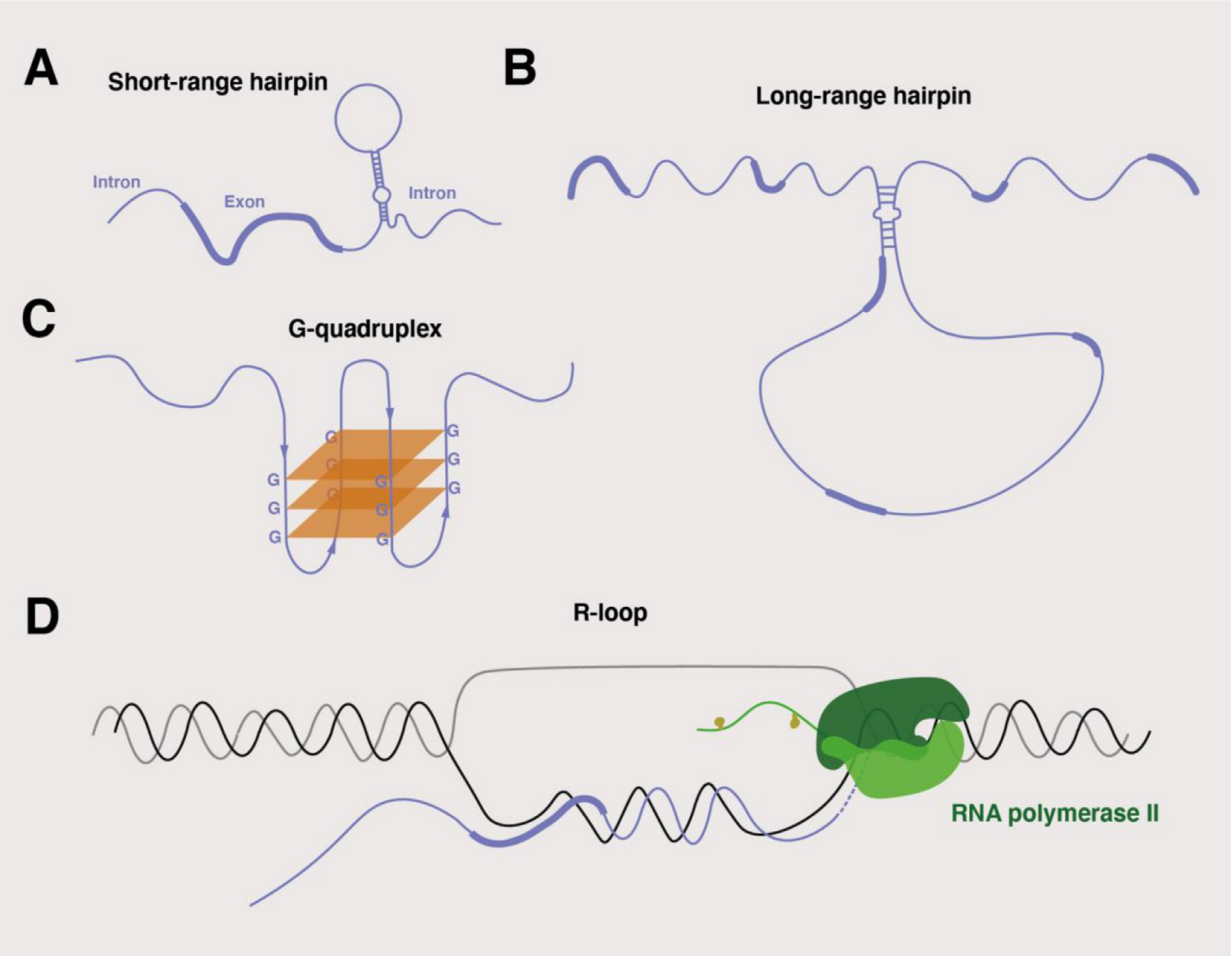
**RNA and DNA-RNA hybrid secondary structures. A.** Hairpin formation in which the stem hybridizes with hydrogen bonds while the loop remains single stranded. **B.** A long range interaction with an imperfect hairpin containing a bulge **C.** A G-quartet is formed by four guanines linked with Hoogsteen hydrogen bonds with each other (shown as squares in brown). Hoogsteen base pairing is a type of non-Watson–Crick base pairing. G-quadruplexes are formed by the stacking of multiple G-quartets. **D.** R-loops are three stranded DNA:RNA hybrid structures that can be formed co-transcriptionally at the template strand. The nascent RNA produced by the RNAPII (**shown in green**) hybridizes with the template strand to form an R-loop structure, while the non-template strand remains single-stranded. Phosphorylation events in the Carboxy-Terminal Domain (CTD) of RNA polymerase II are shown in yellow. In schematics A, B and D thicker lining of the mRNA indicates exonic regions whereas thinner lining indicates intronic regions. (For interpretation of the references to color in this figure legend, the reader is referred to the web version of this article.)

In RNA, intra and intermolecular long-range interactions, including hairpins, pseudoknots, and G-quadruplexes, are commonly observed. Hairpins are composed of a hybridized stem and a single stranded loop (Fig. 1a and b) and can contain mismatches and bulges. Pseudoknots contain nested stem-loop structures, with half of one stem intercalated between the two halves of another stem. G-quadruplex formation is driven by the inherent propensity of guanines to self-assemble, in the presence of monovalent cations, into planar structures known as G-quartets [1]. Each G-quartet is composed of four guanine nucleotides that interact with each other through Hoogsteen hydrogen-bonds. Consecutive runs of guanines (G-tracts) may lead to the formation of consecutive G-quartets that can stack with each other to form G-quadruplex structures (Fig. 1c). Biophysical properties such as the length of intervening loops between consecutive G-runs influence their formation dynamics. In addition, G-quadruplexes can be intramolecular or intermolecular. During transcription, dynamic hybrid structures between DNA and nascent RNA transcripts can be formed, such as R-loops (Fig. 1d) [2]. R-loops are three stranded hybrid structures in which an RNA molecule invades and hybridizes with one DNA strand, while displacing the other. The size of R-loops can range from <100 base pairs to >2000 base pairs [3]. Formation and stabilization of R-loops is particularly favorable when the non-template strand is G-rich, but it can also be promoted by DNA supercoiling, the presence of DNA nicks, and the formation of G-quartets [3], [4]. The impact of R-loop formation, as well as the formation of DNA and RNA G-quadruplexes and other secondary structures, impacts transcript elongation rates and can have a kinetic repercussion on co-transcriptional events involved in RNA processing, such as alternative splicing [5], [6].

A number of methods that probe RNA structures have been developed. Methods such as selective 2′-hydroxyl acylation analyzed by primer extension (SHAPE)-seq [7] and parallel analysis of RNA structure (PARS) [8] were able to identify RNA structures *in vitro,* while more recent methods can deduce structures *in vivo* [9], [10]. For instance, RNA in situ conformation sequencing (RIC-seq) [11] is a powerful new method that enables global detection of intra- and intermolecular RNA–RNA interactions, such as duplexes and long-range loop-loop interactions. Cross-linking immunoprecipitation high-throughput sequencing (CLIP-seq) enables the investigation of protein interactions with RNA molecules [12] from which many variant technologies have emerged. RNA G-quadruplexes can be characterized transcriptome-wide [13], [14] using rG4-seq, which is a modified sequencing method that stalls at RNA G-quadruplexes, enabling identification of RNA G-quadruplexes *in vitro*, and RNA G-quadruplexes have also been visualized *in cellulo* using a specific antibody [15]. Moreover, researchers have developed small molecules, such as carboxy-pyridostatin, a cyanine dye called CyT and Thioflavin T [15], [16], [17], [18], [19], that can shift the equilibrium between the folded and unfolded state of RNA G-quadruplexes and which display preference for RNA over DNA G-quadruplexes. Identification of R-loops has been enabled by usage of specific antibodies [20], [21], [22], [23] and other nuclease-based methods [24], [25].

## 3 RNA polymerase speed and secondary structures

**Fig. 2:**
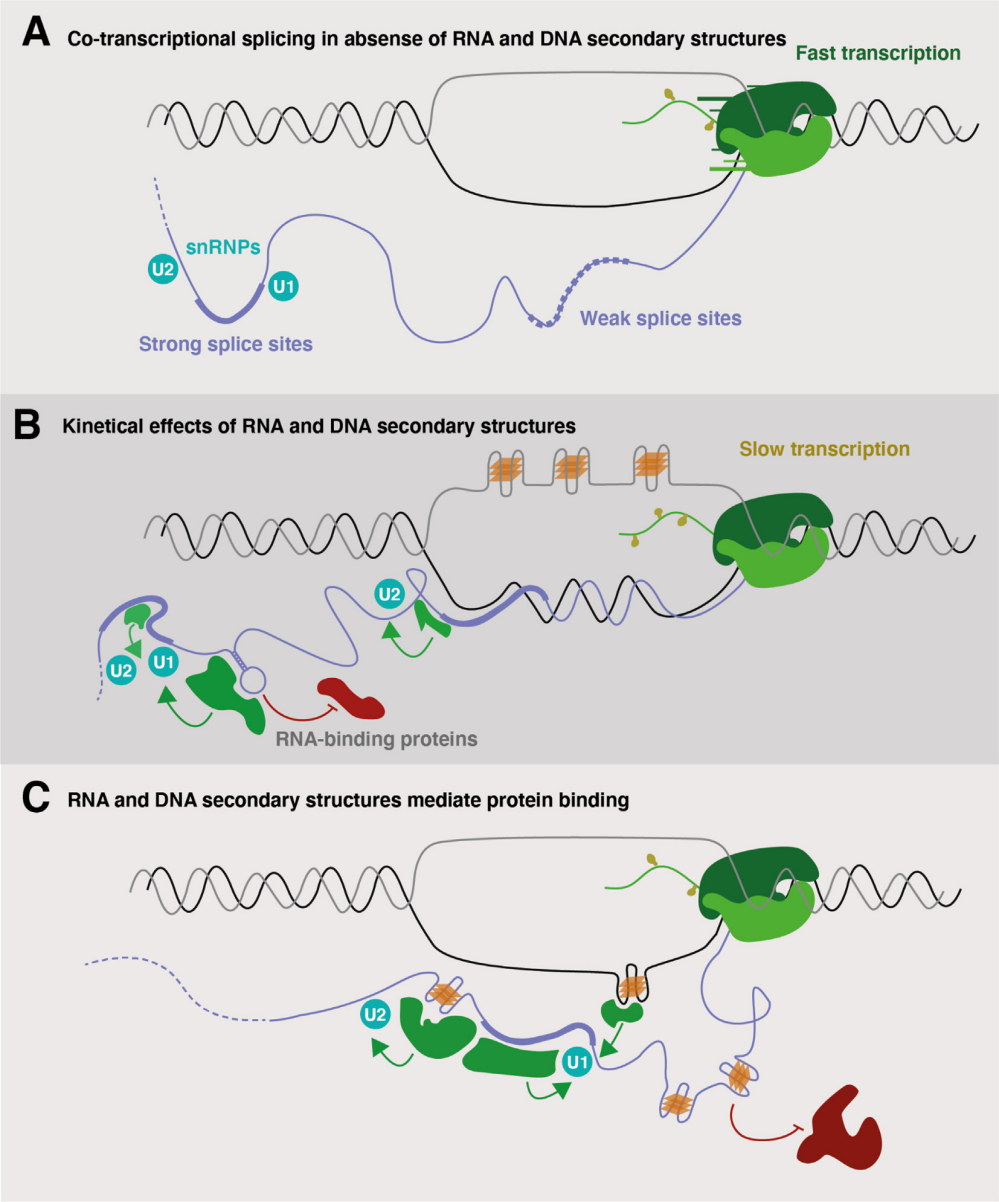
**Mechanisms by which structure formation influences splicing. A.** In the absence of secondary structures, RNAPII elongation rate is higher, which disfavors the recruitment of splicing factors that promote assembly of the spliceosome and exon definition. In this situation exons flanked by weak splice sites may not be recognised, and they are consequently skipped. Exons flanked by strong splice sites can be efficiently recognized by small ribonucleoproteins (snRNPs) U1 and U2, leading to the formation of the pre-spliceosome (complex A) and promoting exon definition and inclusion in the mature mRNA transcripts. **B.** Formation of secondary structures at DNA and RNA can decrease RNAPII elongation speed. For example, during transcription R-loops formed at the 3′ of genes can be stabilized by non-template DNA G-quadruplex formation. Low transcription rates promote exon inclusion by allowing the formation of secondary structures and binding of proteins that can favor the recognition of weak splice sites that would not be recognized otherwise. An RBP that recognizes and binds to the secondary structure is shown in green whereas an RBP whose binding is inhibited by secondary structure formation is shown in red. **C.** RNA secondary structures can modulate mRNA interactions with RBPs either promoting or inhibiting their binding at the mRNA molecule. For example, G-quadruplexes formed at the DNA or RNA level can selectively recruit RBPs to influence splicing outcome. In schematics A, B and C, thicker lining of the mRNA indicates exonic regions whereas thinner lining is indicating intronic regions. The dashed line of mRNA molecules indicates that the length of the transcript can be longer than displayed. (For interpretation of the references to color in this figure legend, the reader is referred to the web version of this article.)

A variety of features are associated with RNAPII speed. For instance, the presence of introns and the length of the first intron are both positively correlated with RNAPII speed [26], while nucleosome formation can reduce RNAPII speed [27], [28]. Regions with high propensity of forming DNA, RNA, or hybrid secondary structures are also associated with RNAPII pausing or slower RNAPII speed (Fig. 2a and b) [29], [30], [31]. Another example of structure remodeling due to slower RNAPII speed is inhibition of hairpin formation due to competition with other alternative structures resulting in reduced binding by stem–loop-binding proteins [30]. In *S. cerevisiae* and *S. pombe*, folding energy and GC content in the transcription bubble have been correlated with RNA polymerase distribution, and RNA structures within nascent transcripts promote forward translocation of the polymerase and limit back-tracking [32]. This indicates how nascent RNA structures can promote the forward movement of an RNA polymerase molecule. Analyses of nascent RNAs have provided evidence that the formation of secondary structures within introns is associated with more efficient co-transcriptional splicing, which is favored under slower transcriptional rates [32], [33]. Taken together, secondary structures will impact several processes, including promoter-proximal pausing, exon recognition, splicing and transcription termination, as they are all influenced by RNAPII speed.

## 4 RNA splicing and secondary structures

Pre-mRNA splicing is a key biological process that enables the removal of introns and the joining of intervening exons, eventually resulting in a mature mRNA molecule. Alternative splicing affects approximately 90–95% of mRNA transcripts in humans [34], [35] and most often occurs co-transcriptionally [33], while for a minority of transcripts it occurs post-transcriptionally [36]. Splicing is a highly conserved mechanism [37] that is pivotal for a number of biological processes such as cell growth, differentiation, immune response, neuronal development [38], [39], [40], while aberrant splicing is implicated in multiple diseases [41] including neurological disorders [42] and cancer [43].

Splicing is mediated through the spliceosome complex which recognizes splice signals, the key members being the 5′ splice site (5′ss), the 3′ splice site (3′ss), and the branch point. The recognition of these consensus sequences is commanded by U1 and U2 small nuclear ribonucleoproteins (snRNPs) and other auxiliary protein factors that are involved in early spliceosomal assembly. Since higher-eukaryotic genes are often interrupted by long introns, early spliceosomal complex assembly over exons recognizes both splice sites during a process commonly known as exon definition [37]. Nevertheless, computational analyses of vertebrate splice sites have shown that the consensus splicing signals only account for approximately half of the information required to accurately define exon/intron boundaries [34], suggesting that other regulatory elements such as RBP sites and secondary structures are crucial for splice site definition. Splice sites with sequences that are substantially different from the consensus signals lead to suboptimal recognition of splice sites (weak splice sites), and are often associated with alternative splicing events. Recent models using deep learning can predict to a large extent splicing events using the primary DNA sequence and can integrate the effects of mutations [44], [45].

Even though the RNA structural code has been less explored [46], it is known that the effects of *cis*-regulatory elements can be modulated by the presence of RNA structures in nascent transcripts and in mature mRNAs [47]. Co-transcriptional transient RNA structure formation can impact splicing through RNAPII pausing and backtracking, which can have a direct kinetic effect over co-transcriptional splicing events [48]. One such example is the human *ATE1* gene, where splicing of two mutually exclusive exons is regulated by competing long-range hairpin structures that span up to 30 kB [49]. Mutations that disrupted each of the secondary structures shift the equilibrium between the two exons indicating direct control of splicing outcome. Reduction of transcription rates can favor further formation of RNA secondary structures [30] and binding of splicing regulatory factors that can increase splicing efficiency therefore allowing the recognition of exons that are flanked by weak splice sites, which would otherwise be skipped [5], [50] (Fig. 2a and b).

## 5 The interplay between RBPs and secondary structures

During the mRNA lifecycle, RBPs regulate to a significant extent diverse transcriptional and post-transcriptional stages including splicing, transportation, translation, stability and degradation. They bind to pre-mRNA molecules in the nucleus and regulate its maturation and transportation to the cytoplasm where they regulate translation and degradation. The number of proteins that can bind to RNA in humans is estimated to be more than 1,500, adding complexity to all the aforementioned programs [51].

RBPs can facilitate or inhibit the recognition of splice sites thereby acting as splicing enhancers or splicing silencers [46], [52], [53]. The majority of RBP motifs are not bound *in vivo* as demonstrated by high-throughput experiments that identify the sites where RBPs bind to endogenous RNAs such as cross-linking immunoprecipitation followed by high-throughput sequencing (CLIP-seq). One possible explanation is that RNA structures provide additional contextual features beyond the primary motif sequences (Fig. 2b and c), and it has also been shown that RNA secondary structure is predictive of binding [54], [55]. Several studies have shown that during pre-mRNA synthesis the formation of RNA structures influences alternative splicing by diverse mechanisms [56], [57], and that local RNA structure formation can impact splicing by modulating the accessibility of core splicing signals [58], [59], [60] as well as RBP binding sites [58], [61], [62].

An example of how RNA secondary structures can dictate the binding of specific RBPs, is provided by MBNL1 and U2AF65 binding to influence exon inclusion in the fifth exon of TNNT2 [63], [64]. MBNL1 favors hairpins and when bound inhibits U2AF65, which favors a linear structure, from binding the polypyrimidine tract resulting in exon skipping. Additional evidence from mice shows that MBNL1 also binds the hairpin structure of exon F in TNNT3. Another example is elF3, which recognizes and binds to hairpin structures at 5′UTR to exert translational activation or repression [65]. Other studies have shown preferential binding of RBPs at RNA G-quadruplex sites, e.g. CNBP, which prevents RNA G-quadruplex structure formation and promotes translation [66] and FMRP, which preferentially binds RNA G-quadruplex structures [66], [67]. Secondary structures and RNA binding proteins have been systematically investigated, enabling the identification of preferences of structured RNA for particular proteins [68], [69]. Interestingly, a recent genetic study showed that G-quadruplex sequences at 5′UTRs are selectively constrained and are enriched for eQTLs, loci containing genetic variants that result in changes of the expression level of a gene, and RBP sites [70].

## 6 Helicases as key regulators of secondary structures

Structure formation is to a large extent modulated by enzymes such as eIF4A and DHX29, that can unwind them, and their importance is demonstrated by their pivotal role in translation initiation [71], [72]. Similarly, the continuous activity of DNA/RNA helicases and ribonucleases H (RNAse H1 and H2) release R-loop structures [3]. Interestingly, R-loops and G-quadruplexes were both found to be unwound by the helicase DHX9 in humans [73]. DHX9 activity protects single-stranded DNA against damage and preserves genomic stability [74]. RNA G-quadruplexes are known to interact with several proteins [70], [75], [76]. For example, the RNA helicase RHAU (also known as DHX36) resolves mRNA G-quadruplexes [77], [78]. One of its targets is a G-quadruplex at the 5′UTR of Nkx2-5 mRNA, and it has been shown that DHX36-mediated G-quadruplex structure unfolding is required for the gene to be expressed [79]. Another DHX36 target is *Gnai2* mRNA, a key regulator of stem cell function and muscle regeneration [78]. DHX36 and DHX9 were also found to modulate translational efficiency by resolving 5′UTR RNA G-quadruplexes [80], while several RBPs such as hnRNP H/F and helicases such as DDX21, DDX17 DDX3X, DDX5 and DDX1 have been found to unwind RNA G-quadruplexes and are also involved in transcription, splicing and translation regulation [81], [82], [83], [84]. Similarly, multiple helicases have been shown to resolve hairpin structures. For instance, UPF1 can resolve RNA hairpins [85], while DDX5 can resolve DNA and RNA G-quadruplexes as well as hairpin structures [86], [87] (Table 1).

**Table 1:** **Important helicases that play a role unwinding RNA and DNA secondary structures. G4s in the table refer to G-quadruplexes.** This a non-exhaustive list of relevant DNA/RNA helicases. Additional examples are reviewed by [92], [93], [94]. Alternative gene names are listed between parenthesis and gene paralogs with homologous functions are separated by “/”.

Gene name	Target	Molecular function	Associated phenotype upon loss of function experiments
*PIF1*	DNA G4	Prevent genome instability associated with DNA G4s and R-loops. [95], [96].	Absence or deficiency of PIF1 increases replication stress and induces DNA damage [95], [96].
ERCC2	DNA G4	XPD is involved in nucleotide excision repair [97]. Evidence suggests that its helicase activity unwinds G4 during transcription [98].	Knock down of XPD results in accumulation of G4s [99].
*BLM*	DNA G4 D-loops Holliday junctions	Unwinds a variety of structures DNA that emerge during DNA replication, recombination and repair [100].	Loss of functions mutations leads to Bloom syndrome [101]. Absence of BLM is associated with genome instability and excess of sister chromatid exchange events at G4 loci [102].
*WRN*	DNA G4 R-loops	Prevents genome instability associated with DNA G4s and R-loops [103], [104].	WRN loss of function leads to accumulation of G4s and expression changes associated with G4-containing promoters [105].
*DHX9 (DDX9)*	RNA G4 R-loops H-DNA	Involved in DNA replication, transcription and translation [106]. Resolves R-loop and H-DNA structures to promote genomic stability [107], [108], [109]. Unwinds RNA G4s to control translation [80].	Absence of DHX9 promotes back-splicing events and induce translational repression of transcripts containing inverted-repeats Alu elements [110].
*DHX36*	DNA/RNA G4	Activates transcription by resolving DNA G4s at promoters [111], [112]. Unwinds RNA G4s to control translation [80], [113] and miRNA biogenesis [114].	Formation of stress granules and increases protein kinase R (PKR) phosphorylation [113]. Reduced telomerase efficiency and shorter telomeres [115]. Higher UV sensitivity due to lack of p53 expression [116].
*DDX5/DDX17*	DNA/RNA G4 RNA Hairpins	Paralogues that encode for helicases that resolve RNA hairpins and G4s, having a regulatory role in alternative splicing and translation [84], [86], [117]. DDX5 also resolves DNA G4s that control gene transcription [87].	Knock out leads to mouse embryonic lethality [118]. DDX5/DDX17 absence impairs splicing and miRNA biogenesis during neuronal differentiation [119].
*DDX21*	RNA G4 R-loops	Involved in ribosomal RNA biogenesis and anti-viral immune response [120], [121], [122].	DDX21 knock down results in increased expression of genes with G4 motifs in their 3′UTR [83].
*DDX1*	RNA G4	Converts RNA G4 into R-loops [81].	DDX1 deficiency impairs class switch recombination in B cells [81]
*DDX2A/DDX2B* (EIF4A1/EIF4A2)	RNA hairpins RNA G4	Paralogues that encode for the two subunits of the eukaryotic translation initiation factor 4A (eIF4A). These helicases resolve RNA hairpins and G4s located at the 5′-UTR, which has an impact on mRNA translation efficiency.	DDX2A plays an essential role in spermatogenesis, whereas DDX2B is essential for mouse viability [123].
*DDX41*	R-loops	Resolves R-loops that emerge during transcription [124].	R-loop accumulation and genomic instability due to knock down of DDX41 [124].
*DDX39B* (*UAP56*)	R-loops	Spliceosomal helicase with roles in the removal of R-loops [125].	R-loop accoumlaton, genomic instability and replication fork stalling [125].
*SETX*	R-loops	Senataxin removes R-loops to maintain genome integrity [126].	Knock down of Senataxin results in an increase in R-loops downstream of the poly(A) signal [127].
*AQR* (*EMB4*)	R-loops	Intron-binding spliceosomal factor with helicase activity that contributes to R-loop removal [128], [129].	Genome instability and deficiency in co-transcriptional gene silencing pathways mediated by small RNAs [129], [130].

The cellular mechanisms mediating the stabilization and resolution of RNA secondary structures remain incompletely understood, as are the interactions between secondary structures and protein complexes. In addition, the effect of perturbing these mechanisms and their relevance to disease progression is unclear. High throughput screens coupled with short hairpin RNAs (shRNAs) or CRISPR-based technologies have enabled systematic interrogation of the roles of diverse proteins, such as RBPs, helicases, and topoisomerases [88], [89], [90], [91]. Furthermore, mutational analysis with CRISPR-Cas9 could be used to study the effects of secondary structure disruption *in vivo* or *in cellulo*. CRISPR-induced mutations that destroy the secondary structure motifs, for example the G-runs of G-quadruplexes or the stem sequence of hairpins, but leave other regulatory sequences such as RBP motifs unchanged, could advance the understanding of how secondary structures determine gene expression.

## 7 G-quadruplexes as regulators of alternative splicing

G-quadruplex sequences are enriched at promoters and they have been extensively studied in this context [131]. Additionally, G-quadruplexes have been related to splicing, 3′ processing, transcription termination, RNA localization and translation regulation [76]. Interestingly, it has been shown that G-quadruplex sequences have a high enrichment in the proximity of both 3′ and 5′ splice sites across a wide range of species. The effect is more pronounced at the non-template strand, suggesting that the G-quadruplexes are formed primarily by the RNA and that they may favor or block the binding of RBPs [132].

One of the first exemplary cases of RNA G-quadruplex mediated regulation of alternative splicing was found in the hTERT gene, which encodes for the catalytic subunit of the telomerase enzyme, and one of its exon skipping events is promoted by the stabilization of intronic G-quadruplexes [133]. Gomez and colleagues hypothesized that RNA G-quadruplex formation can prevent RBP binding to intronic enhancers, leading to exon skipping. However, based on different functional assays, RNA G-quadruplex formation has also been proposed to promote RBP binding to splicing regulatory elements [134], [135], [136]. Since G-quadruplex-dependent splicing events were often demonstrated by introducing mutations at G-quadruplex motifs, it was unclear from these results whether the G-quadruplex structure or the linear form of these G-rich sequences act as a splicing enhancer. To disentangle these effects, Huang and colleagues showed that mutations that prevent intronic G-quadruplex formation but keep G tracts intact, led to exon exclusion of an alternative exon in the *CD44* gene [137]. Since the CD44 intronic G-quadruplex motif sequence can be bound by two RBPs that have the opposite effect on exon exclusion, RNA G-quadruplex formation may function as a switch to promote the binding of one RBP over the other [138]. In another recent study where the role of wild-type and mutated G-quadruplex sequences in alternative splicing was tested using a minigene, it was also shown that the presence of an RNA G-quadruplex favors exon inclusion [132], consistent with the aforementioned findings. There is also evidence of an interplay between RNA G-quadruplex stabilization and specific binding proteins such as HNRNP H/F [116], [137] and HNRPU [139] and recent studies suggest that RNA G-quadruplex formation can modulate *in vitro* RBP binding to mRNA molecules [66].

The genome-wide effect of RNA G-quadruplex formation over splicing factor binding remains unclear. High-throughput screening of chemical compounds via dual-color splicing reporters has identified two small molecules, emetine and cephaeline, that disrupt RNA G-quadruplex formation [140]. Genome-wide evaluation of emetine effects on alternative splicing showed substantial alternative splicing changes after treatment, with nearly 60% being exon skipping events. It was also shown that multiple RBPs colocalize with G-quadruplex motifs flanking splice junctions, suggesting an interplay between RBP binding and RNA G-quadruplex structure formation, which was further corroborated by loss of function experiments followed by RNA-seq, identifying consistent associations for 36 RBPs [132], [137].

## 8 Hairpins enable long range RNA interactions during splicing

Long range interactions are important for splicing modulation [141], and they are more enriched at weak alternative acceptor splice sites [142]. Some long range interactions can span several kilobases and can bring in proximity otherwise distant splice sites. One of the best-characterized examples of regulation of splicing through RNA structures can be found in *D. melanogaster* for the *DSCAM* gene, where RNA-RNA interactions, mediated through multiple structures, regulate the selection of exons within arrays of mutually exclusive exons [143], [144]. In this case, RNA looping can bring splicing elements situated thousands of bases away from each other into close proximity.

Hairpins may also directly affect exon skipping events by a mechanism known as “looping-out”, whereby inter-intronic base-pairing RNA interactions can loop out exons to promote their skipping [56]. This mechanism is supported by the enrichment of conserved complementary sequences present in intronic regions flanking exon skipping events [145]. Moreover, the artificial introduction of self-complementary regions across exons suppresses exon inclusion in yeast, suggesting a causal relationship between hairpins and exon skipping [146]. Interestingly, the expansion of self-complementary regions is related to the primate-specific Alu retrotransposon, which is enriched in regions flanking alternative exons, suggesting a role in splicing regulation [147]. During back-splicing, an unconventional splicing mechanism, the second nucleophilic attack is performed over an upstream 3′ splice leading to circular RNA (circRNAs) products. circRNAs are particularly abundant in the brain and RNA structures that favor back-splicing are often derived from complementary intronic sequences associated with Alu elements [148]. In zebrafish, hairpin formation between dinucleotide repeats that co-occur at opposite boundaries of an intron, mediate splicing without U2AF2, which is a major component of the spliceosome [149].

The formation of RNA structures can also enhance RBP regulatory range by bringing distal regulatory elements in close proximity with their exon targets [150]. This can be particularly important for RBFOX2 regulated exons since more than half of RBFOX2-binding sites are found over 500 bp away from any annotated exons, and it has been shown that long-range RNA hairpin formation is necessary for the regulatory effect of distal binding sites [151]. It has also been shown that hairpin formation can influence splicing regulatory protein binding, with enhancers and silencers having a stronger effect when present in the loop relative to the stem [52], [54], suggesting that RBP binding is inhibited at the stem [58], [61]. In an elegant set of experiments, it was shown that in the case of *FGFR2*, the formation of a hairpin structure is required for efficient splicing from two mutually exclusive exons and its splicing effect is not dependent on its primary nucleotide composition as shown using minigene assays [152].

The fibronectin EDA exon is controlled by seven hairpins and a key exonic splicing enhancer is found in the loop of one of the hairpins, which is in turn bound by splicing regulatory proteins such as SRSF1 [153], [154]. Other examples include a hairpin which modulates the inclusion of the alternative exon 6B of the β-tropomyosin transcript in chicken [155]. It was also shown that a mutation in PS2 that deletes or destabilizes a hairpin in exon 5, results in higher levels of exon inclusion [156]. Importantly, the formation of hairpin structures could be dynamic and due to environmental changes, an example being temperature-dependent formation of a hairpin that controls splicing of APE2 gene in yeast [157]. In addition, alternatively spliced exons display an enrichment for secondary structures and evolutionary conservation of many of these structures indicates their important regulatory functions [57]. This is exemplified by conservation of secondary structures over the primary nucleotide sequence such as a conserved hairpin structure in RB1CC1 [57]. Advances in long-read RNA sequencing technologies will enable improved detection of long-range interactions and their impact in the regulation of alternative splicing events.

## 9 The role of RNA structures on RNA stability and decay

The half-life and decay rates of mRNA transcripts in human cells influence protein expression levels. A number of features determine transcript stability including GC content, transcript length, polyA tail length, RBP sites, microRNA binding sites, and mRNA secondary structures [158], [159], [160], [161], [162], [163]. Structural features of mRNAs dictate to a large extent mRNA half-life with transcripts that have a structured coding sequence showing higher expression levels [159]. Hairpins in mRNA transcripts can result in increased stability [163], [164], [165], such as when found at the 3′UTR near mRNA cleavage sites. The accessibility of microRNA sites influences mRNA half-life and secondary structure formation can change the microRNA binding efficiency [166]. For example, the introduction of a hairpin in the 5′UTR of a transcript, results in substantial increases in gene expression [167], [168]. Constitutive decay elements are RNA motifs that mediate the destabilization and degradation of mRNA molecules, and contain a hairpin sequence [169] at which Roquin proteins bind to induce the decay of the transcript [170].

Massively parallel reporter assays are high-throughput technologies that enable rapid measurements of thousands of sequences for their regulatory activity and have received widespread adoption in recent years [171], [172], [173], [174]. Multiple variants of this technology have been implemented to study a plethora of gene regulatory elements, including promoters, enhancers, 5′ UTRs, and 3′ UTRs, by placing synthetic sequences in the appropriate location relative to a reporter gene. In this case massively parallel reporter assay experiments have shown that its destabilizing effects increase as a function of the hairpin length [165].

## 10 Secondary structures in translation

**Fig. 3:**
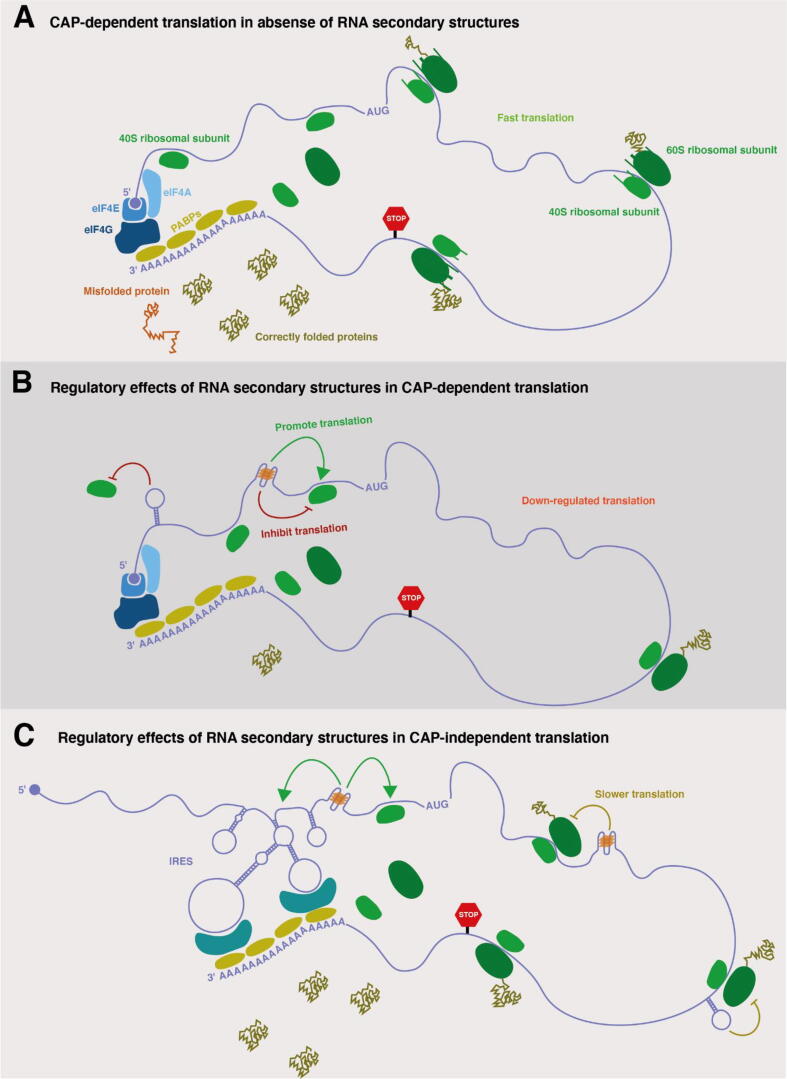
**Mechanisms by which RNA structure formation influences translation. A.** During cap-dependent translation, translation initiation factors (blue proteins) recognize the mRNA 5′ cap structure (purple circle) and bridge its interaction with the 3′ polyA tail, through polyA binding proteins (PABPs). During translation several helicases actively unwind the mRNA, which could remove secondary structures. This could lead to faster ribosome speeds, which may result in protein misfolding. **B.** Cap-dependent translation can be regulated by the dynamic formation of secondary structures in the 5′ UTR. Hairpin formation can limit the binding of the ribosome and translation initiation factors, thereby repressing protein translation. The presence of G-quadruplexes in the 5′ UTR may inhibit translation directly, activate upstream ORFs, or promote translation. **C.** Cap-independent translation can take place in the presence of IRESs, which require highly structured 5′UTR domains that indirectly interact with PBAPs to promote mRNA circularisation. Some IRES structures can be activated by RNA G-quadruplex formation. Further formation of RNA secondary structures across the ORF can limit the translation speed and favor a step-by-step modular folding. Additional details on Cap-dependent and Cap-independent mechanisms are comprehensively reviewed at [234]. (For interpretation of the references to color in this figure legend, the reader is referred to the web version of this article.)

Translation can be divided into four phases, initiation, elongation, termination and ribosome recycling [175], [176]. Initiation is the rate limiting and most regulated step, consisting of several complex programs. The regulation of translation directly impacts protein levels with most regulatory mechanisms affecting the rate-limiting initiation step [177], [178], [179]. The multifarious effects of translational control can be observed across biological processes including development, differentiation, functions of the nervous system and disease [177], [180]. Initiation can be either cap-dependent or cap-independent [181], [182]. Cap-dependent translation is the most frequently used in eukaryotes and starts with the binding of eIF4E to the mRNA cap. The most common cap-independent initiation mechanism, often utilized by viral RNAs, involves an internal ribosome entry site (IRES) of structured mRNA. IRES structures can recruit ribosomal subunits and eukaryotic initiation factors [183]. RNA molecules fold in complex configurations with the presence of RNA secondary structures in the 5′UTR being a major determinant of the rate of translation (Fig. 3a and b) [184], [185], [186]. Moreover, the ribosome itself is a major remodeler of RNA structure [187]. Lower translation rates can not only limit protein abundance, but can also enable correct co-translational protein folding [188], [189]. In addition, secondary structures can influence the recognition of the IRESs (Fig. 3c).

Although the vast majority of eukaryotic translation start sites have an AUG codon, often the first AUG codon is bypassed, resulting in usage of more distal AUG codons and alternative protein isoforms. This process is referred to as leaky scanning, with a proportion of ribosomes initiating translation from downstream start codons. Leaky scanning and translational efficiency are influenced by the presence of secondary structures [8], [190], [191], [192]. Moreover, there is a large proportion of suboptimal start sites that do not contain the canonical start codon. Microsatellite expansions can cause non-AUG initiation [193]. These non-AUG start sites are often associated with alternative translation start [194], [195]. Ribosome profiling is one of the primary methods of identifying the occupancy of elongating ribosomes on mRNAs, therefore providing a direct readout of ribosome decoding rates [176].

Secondary structures can conceal or expose binding sites for translation regulators, and it has been shown that certain RBPs bind preferentially at structured RNA while others have a preference for linear forms [196]. Moreover, formation of secondary structures can change the distance between translation-associated motifs, an example being the distance between the stem-loop and the cap [197]. Secondary structure formation can also promote cap-independent translation, and the disruption of an IRES hairpin can in turn reduce translation efficiency in viral [198], [199] and eukaryotic [200] mRNAs.

Riboswitches are components of mRNA molecules that can bind a small molecule and directly control gene expression through RNA conformational changes, without proteins being involved. They are found in both prokaryotes and eukaryotes, with most discovered riboswitches being present in bacteria and archaea [201]. The aptamer is a receptor for a small molecule, and it is usually located in the 5′UTR of a mRNA where it forms a secondary structure that binds to the small molecule. The expression platform is the regulatory domain of the riboswitch and it modulates gene expression upon binding of the small molecule. Riboswitches have been found to regulate a number of processes including initiation of translation [202], mRNA decay [203], transcription termination [204] and splicing [205], [206]. For instance, in *E. coli* the lysine riboswitch when lysine is present it restricts translation initiation and also exposes RNase E cleavage sites [203].

RNA structures can directly interact with the translational machinery and influence the recognition of the translation start [207]. Note that the interaction is complicated by the fact that the translational machinery can unwind and remodel RNA structures [187]. There is also decreased translational efficiency at highly structured 5′UTRs [80], [208]. For example, in the case of *BRCA1*, a tumor suppressor gene, a longer 5′UTR isoform is expressed only in breast cancer cells, resulting in a 10-fold decrease of translational efficiency due to the formation of a stable complex secondary structure [208]. Finally, the interplay between RNA structure formation and unwinding influences ribosome initiation, scanning and elongation. Therefore, secondary structures can account for differences between mRNA and protein levels [209].

## 11 Hairpins enable long range RNA interactions in translation initiation

Early studies indicated that hairpin formation can influence translation efficiency [210]. Hairpins with high thermal stability upstream of the translation start site resulted in reduced translation by up to 85–95%, whereas hairpin formation downstream of an AUG at specific positions resulted in an increase in translation rate by facilitating recognition of initiator codons by ribosomes [211], [212]. Stem length and GC content, both of which increase thermal stability, inhibit translation, while more distant hairpins have a smaller inhibitory effect [213]. Other studies have also indicated that both the GC content of the stem and the positioning of the hairpin relative to the translation start site dramatically influence the translation efficiency [207].

Hairpins at the 5′UTR of ferritin-H and ferritin-L mRNAs act as an iron-responsive element controlling iron levels and are highly dynamic response elements to environmental changes [214]. Another example is a hairpin structure in the c-JUN 5′ UTR which is recognized by eIF3 and is required for initiation of translation [215]. Another study generated a library of half a million 50 bp long 5′UTRs and identified hairpin structures to negatively impact protein levels, especially those with longer stems and shorter loops [216].

## 12 G-quadruplexes in translation initiation

RNA G-quadruplexes are enriched at 5′UTRs (Huppert et al. 2005) where they show a higher frequency at the template strand, suggesting a relative depletion of G-quadruplexes at the RNA level [217]. There is also a difference in the density of G-quadruplexes, with the highest density being found within 50 bp of the start of the 5′UTR and a declining frequency moving away from it [217]. It has been shown that G-quadruplexes in the 5′UTR of mRNAs are inhibitory elements [218], and several studies have since shown that G-quadruplexes at the 5′UTR interfere with the recognition by ribosomes [17], [219], [220], [221], [222], [223]. Specifically, experiments involving luciferase plasmid vectors indicate that G-quadruplexes inhibit expression across 5′UTR regions, perhaps by interfering with ribosome scanning. However, in many of these experiments the researchers used controls where guanines had been substituted for uracils, potentially also interfering with RBP binding sites and the GC content [218], [219].

It has also been shown that G-quadruplexes at 5′UTRs of eukaryotic genes can promote translation by favoring recognition of the IRES [224], [225], [226], [227]. In FGF-2, a gene that is associated with tissue development and repair, a G-quadruplex motif together with two hairpin sequences are found within the IRES, and they promote translation in a cap-independent translational program [225]. A G-quadruplex site in the RBP FMRP is a binding site for the protein itself, and it has been suggested that it could in this way control both its own expression levels [228] and its mRNA splicing [134]. In VEGF, an RNA G-quadruplex was shown to be essential for IRES-mediated translation initiation [227], [229], [230]; however other studies have contended its role and provided evidence for inhibitory functions [231], [232].

A study that used massively parallel reporter assays to investigate mRNA translation found that G-quadruplexes in the 5′UTR act as translational inhibitors, and that knockdown of G-quadruplex resolving helicases aggravated these phenotypes [233]. It was also found that RNA G-quadruplex formation could promote the usage of an upstream translation start site by slowing down the pre-initiation complex scanning [80]. The role of secondary structures was systematically explored in a high-throughput experiment where half a million 50 bp randomly generated 5′UTRs were synthesized and tested in yeast. The results showed that several secondary structures, including RNA G-quadruplexes and hairpins, are important contributors to expression levels [216]. RNA G-quadruplexes can either restrict or promote the recognition by ribosomes and even though there are more studies indicating inhibitory functions, it is not clear which effect is more widespread and what features determine if the G-quadruplex will restrict or promote ribosomal recognition.

## 13 Splicing and translation associated secondary structures in disease

Regions that are predisposed to secondary structure formation, such as G-quadruplexes have an excess of germline and somatic mutations [235], [236]. The functional role of these structures is supported by the observation that eQTLs are enriched at G-quadruplexes within 5′UTRs and splicing quantitative trait loci (sQTLs) are enriched at G-quadruplex motifs flanking splice sites [70], [132]. The accumulation of R-loops is also associated with genomic instability [237], [238], [239], [240] As secondary structure formation modulates diverse processes including splicing and translation initiation, changes in the mRNA structure have been associated with and can result in human disease.

Mutations of alternative splicing factors can lead to R-loop accumulation, which may compromise genomic stability and be relevant in the context of cancer pathogenesis [241], [242]. RNA splicing perturbation by expression of *U2AF1* or *SRSF2* mutants, mutations that are commonly observed in myelodysplastic syndrome, results in the accumulation of R-loops [243]. In the *MAPT* gene, also known as tau, in the interface between exon 10 and intron 10, there is a hairpin structure which can mask the splice site [244], [245] and DDX5 was found to be involved in the resolution of this hairpin structure controlling splicing of MAPT (tau) exon 10 [86]. Mutations at the hairpin result in its destabilization, causing inclusion of exon 10 due to increased association with U1 snRNP [244] and results in higher prevalence of neurodegeneration. Hairpin sequences were also identified in the 5′UTR of other transcripts including the amyloid precursor protein [246] and α-synuclein [247], indicating the importance of structure-mediated control of expression levels. In spinal muscular atrophy, a stem-loop RNA structure overlaps with the 5′ splicing site of exon 7 of *SMN2* and interference with the structure formation is a therapeutic target against the spinal muscular atrophy molecular phenotype [248]. Sulovari et al. showed that variable number tandem repeats were particularly enriched at Alu elements and found an association between genes differentially spliced or expressed between human and chimpanzee brains [249].

RNA G-quadruplex structures have been identified in several cancer genes, including *TP53* and *TERT*, where they can modulate splicing and protein isoforms [133], [135]. In CD44 an RNA G-quadruplex in intron 8 functions as a splicing enhancer with roles in the control of the epithelial–mesenchymal transition [137], a process that is important for cancer metastasis [250]. One of the canonical translation initiation factors, elF4A, is a DEAD-box RNA helicase that can unwind secondary structures, including RNA G-quadruplexes, and its activity is correlated with the number of secondary structures in the 5′UTR [251]. Perturbation of elF4A can contribute to oncogenesis as it results in formation of RNA G-quadruplexes in the 5′UTRs of mRNAs targeted by elF4A, including many oncogenes, transcription factors, and epigenetic regulators [252].

The expansion of microsatellite repeats at 5′UTRs has been associated with aberrant translation and has been implicated in multiple disorders [193], [253]. The mechanisms involve the formation of secondary structures that interfere with translation and repeat-associated non-AUG translation. One of the most well-studied examples is the expansion of the hexanucleotide GGGGGC in the first intron of the *C9orf72* gene which results in frontotemporal dementia (FTD) and amyotrophic lateral sclerosis (ALS). These repeats form different secondary structures including G-quadruplexes, R-loops and hairpins [254], [255], [256] which leads to aborted transcription at the repeat site [254]. Expansion of these repeats results in repeat-associated non-AUG translation and the generation of toxic dipeptide proteins [257], while reducing DHX36 levels in cells derived from C9orf72-linked ALS patients results in reduced dipeptide protein burden due to the formation of RNA G-quadruplexes [258]. In ALS and FTD, Nucleolin binds to the G-quadruplex forming hexanucleotide repeat, resulting in its mislocalization in the cell [254]. In addition, a number of other proteins associated with the ALS pathology such as TDP-43, FUS/TLS, hnRNPA1, hnRNPA2B1, hnRNPA3 and EWSR1 interact with the RNA G-quadruplex [259], [260], [261], [262], [263], [264]. Encouragingly, G-quadruplex binding small molecules ameliorate the pathologies associated with ALS and FTD in model systems, indicating that RNA G-quadruplexes can pose as a therapeutic target [265]. Beta-amyloid precursor protein cleaving enzyme 1 (BACE1) encodes a protein that cleaves amyloid precursor protein resulting in the generation of amyloid-beta peptide, the accumulation of which is a hallmark of Alzheimer’s disease [266]. An RNA G-quadruplex in exon 3 of BACE1 modulates splicing by inhibiting the binding of hnRNP H, thereby promoting a shorter isoform without the proteolytic activity that creates the neurotoxic peptide [267]. ADAM-10 is also associated with Alzheimer’s disease due to its anti-amyloidogenic activity and a RNA G-quadruplex in its 5′UTR represses its expression [268].

## 14 Concluding remarks

RNA secondary structures are pervasive, interact with RNA binding proteins and are linked to a large number of important functions, including transcription, splicing and translation. Even though the functional importance of secondary structures has been repeatedly demonstrated, the contribution of RNA structures in these processes remains incompletely understood due to the difficulties in identifying dynamic RNA structures and their mechanisms of action. High-throughput technologies enable the systematic investigation of RNA secondary structures and the design of experiments to quantify their contribution in transcription, splicing and translation enables directly testing their mechanisms of action. New methods to dynamically identify RNA secondary structures are gradually revealing their widespread and diverse contributions in gene regulation. However, it remains difficult to capture their dynamic changes across cellular conditions and their interplay with proteins. The degree to which RNA secondary structure formation is influenced by the tissue and cell type remains largely unstudied. The availability of large scale single cell assays will enable the investigation of associations between secondary structures, the presence of various sequence motifs, and expression levels of RBPs across different cell types. Even more interesting could be the combination of single cell technologies with different small molecules that stabilize specific structures.

## Declaration of Competing Interest

The authors declare that they have no known competing financial interests or personal relationships that could have appeared to influence the work reported in this paper.

## References

[1] Gellert M., Lipsett M.N., Davies D.R. (1962). Helix formation by guanylic acid. Proc Natl Acad Sci U S A.

[2] Crossley M.P., Bocek M., Cimprich K.A. (2019). R-Loops as Cellular Regulators and Genomic Threats. Mol Cell.

[3] Santos-Pereira J.M., Aguilera A. (2015). R loops: new modulators of genome dynamics and function. Nat Rev Genet.

[4] Duquette M.L., Handa P., Vincent J.A., Taylor A.F., Maizels N. (2004). Intracellular transcription of G-rich DNAs induces formation of G-loops, novel structures containing G4 DNA. Genes Dev.

[5] Dujardin G., Lafaille C., de la Mata M., Marasco L.E., Muñoz M.J., Le Jossic-Corcos C. (2014). How slow RNA polymerase II elongation favors alternative exon skipping. Mol Cell.

[6] Nieto Moreno N., Giono L.E., Cambindo Botto A.E., Muñoz M.J., Kornblihtt A.R. (2015). Chromatin, DNA structure and alternative splicing. FEBS Lett.

[7] Lucks J.B., Mortimer S.A., Trapnell C., Luo S., Aviran S., Schroth G.P. (2011). Multiplexed RNA structure characterization with selective 2’-hydroxyl acylation analyzed by primer extension sequencing (SHAPE-Seq). Proc Natl Acad Sci U S A.

[8] Kertesz M., Wan Y., Mazor E., Rinn J.L., Nutter R.C., Chang H.Y. (2010). Genome-wide measurement of RNA secondary structure in yeast. Nature.

[9] Spitale R.C., Flynn R.A., Zhang Q.C., Crisalli P., Lee B., Jung J.-W. (2015). Structural imprints in vivo decode RNA regulatory mechanisms. Nature.

[10] Ding Y., Tang Y., Kwok C.K., Zhang Y., Bevilacqua P.C., Assmann S.M. (2014). In vivo genome-wide profiling of RNA secondary structure reveals novel regulatory features. Nature.

[11] Cai Z., Cao C., Ji L., Ye R., Wang D., Xia C. (2020). RIC-seq for global in situ profiling of RNA–RNA spatial interactions. Nature.

[12] Licatalosi D.D., Mele A., Fak J.J., Ule J., Kayikci M., Chi S.W. (2008). HITS-CLIP yields genome-wide insights into brain alternative RNA processing. Nature.

[13] Kwok C.K., Marsico G., Sahakyan A.B., Chambers V.S., Balasubramanian S. (2016). rG4-seq reveals widespread formation of G-quadruplex structures in the human transcriptome. Nat Methods.

[14] Zhao J, Chow EY-C, Yeung PY, Zhang QC, Chan T-F, Kwok CK. rG4-seq 2.0: enhanced transcriptome-wide RNA G-quadruplex structure sequencing for low RNA input samples n.d. https://doi.org/10.1101/2022.02.10.479665. 10.1186/s12915-022-01448-3 PMC9661767 36372875

[15] Biffi G., Di Antonio M., Tannahill D., Balasubramanian S. (2014). Visualization and selective chemical targeting of RNA G-quadruplex structures in the cytoplasm of human cells. Nat Chem.

[16] Di Antonio M., Biffi G., Mariani A., Raiber E.-A., Rodriguez R., Balasubramanian S. (2012). Selective RNA versus DNA G-quadruplex targeting by in situ click chemistry. Angew Chem Int Ed Engl.

[17] Gomez D., Guédin A., Mergny J.-L., Salles B., Riou J.-F., Teulade-Fichou M.-P. (2010). A G-quadruplex structure within the 5’-UTR of TRF2 mRNA represses translation in human cells. Nucleic Acids Res.

[18] Xu S., Li Q., Xiang J., Yang Q., Sun H., Guan A. (2016). Thioflavin T as an efficient fluorescence sensor for selective recognition of RNA G-quadruplexes. Sci Rep.

[19] Xu S., Li Q., Xiang J., Yang Q., Sun H., Guan A. (2015). Directly lighting up RNA G-quadruplexes from test tubes to living human cells. Nucleic Acids Res.

[20] Ginno P.A., Lott P.L., Christensen H.C., Korf I., Chédin F. (2012). R-loop formation is a distinctive characteristic of unmethylated human CpG island promoters. Mol Cell.

[21] Chen L., Chen J.-Y., Zhang X., Gu Y., Xiao R., Shao C. (2017). R-ChIP Using Inactive RNase H Reveals Dynamic Coupling of R-loops with Transcriptional Pausing at Gene Promoters. Mol Cell.

[22] Boguslawski S.J., Smith D.E., Michalak M.A., Mickelson K.E., Yehle C.O., Patterson W.L. (1986). Characterization of monoclonal antibody to DNA.RNA and its application to immunodetection of hybrids. J Immunol Methods.

[23] Sanz L.A., Chédin F. (2019). High-resolution, strand-specific R-loop mapping via S9.6-based DNA-RNA immunoprecipitation and high-throughput sequencing. Nat Protoc.

[24] Wulfridge P., Sarma K. (2021). A nuclease- and bisulfite-based strategy captures strand-specific R-loops genome-wide. Elife.

[25] Yan Q., Shields E.J., Bonasio R., Sarma K. (2019). Mapping Native R-Loops Genome-wide Using a Targeted Nuclease Approach. Cell Rep.

[26] Muniz L., Nicolas E., Trouche D. (2021). RNA polymerase II speed: a key player in controlling and adapting transcriptome composition. EMBO J.

[27] Churchman L.S., Weissman J.S. (2011). Nascent transcript sequencing visualizes transcription at nucleotide resolution. Nature.

[28] Bintu L., Ishibashi T., Dangkulwanich M., Wu Y.-Y., Lubkowska L., Kashlev M. (2012). Nucleosomal elements that control the topography of the barrier to transcription. Cell.

[29] Sun L., Fazal F.M., Li P., Broughton J.P., Lee B., Tang L. (2019). RNA structure maps across mammalian cellular compartments. Nat Struct Mol Biol.

[30] Saldi T., Fong N., Bentley D.L. (2018). Transcription elongation rate affects nascent histone pre-mRNA folding and 3′ end processing. Genes Dev.

[31] Zhang J., Landick R. (2016). A Two-Way Street: Regulatory Interplay between RNA Polymerase and Nascent RNA Structure. Trends Biochem Sci.

[32] Turowski T.W., Petfalski E., Goddard B.D., French S.L., Helwak A., Tollervey D. (2020). Nascent Transcript Folding Plays a Major Role in Determining RNA Polymerase Elongation Rates. Mol Cell.

[33] Saldi T., Riemondy K., Erickson B., Bentley D.L. (2021). Alternative RNA structures formed during transcription depend on elongation rate and modify RNA processing. Mol Cell.

[34] Wang E.T., Sandberg R., Luo S., Khrebtukova I., Zhang L., Mayr C. (2008). Alternative isoform regulation in human tissue transcriptomes. Nature.

[35] Pan Q., Shai O., Lee L.J., Frey B.J., Blencowe B.J. (2008). Deep surveying of alternative splicing complexity in the human transcriptome by high-throughput sequencing. Nat Genet.

[36] Vargas D.Y., Shah K., Batish M., Levandoski M., Sinha S., Marras S.A.E. (2011). Single-molecule imaging of transcriptionally coupled and uncoupled splicing. Cell.

[37] Keren H., Lev-Maor G., Ast G. (2010). Alternative splicing and evolution: diversification, exon definition and function. Nat Rev Genet.

[38] Irimia M., Blencowe B.J. (2012). Alternative splicing: decoding an expansive regulatory layer. Curr Opin Cell Biol.

[39] Kalsotra A., Cooper T.A. (2011). Functional consequences of developmentally regulated alternative splicing. Nat Rev Genet.

[40] Bell L.R., Maine E.M., Schedl P., Cline T.W. (1988). Sex-lethal, a Drosophila sex determination switch gene, exhibits sex-specific RNA splicing and sequence similarity to RNA binding proteins. Cell.

[41] Scotti M.M., Swanson M.S. (2016). RNA mis-splicing in disease. Nat Rev Genet.

[42] Feng D., Xie J. (2013). Aberrant splicing in neurological diseases. Wiley Interdiscip Rev RNA.

[43] Bonnal S.C., López-Oreja I., Valcárcel J. (2020). Roles and mechanisms of alternative splicing in cancer — implications for care. Nat Rev Clin Oncol.

[44] Jaganathan K., Kyriazopoulou Panagiotopoulou S., McRae J.F., Darbandi S.F., Knowles D., Li Y.I. (2019). Predicting Splicing from Primary Sequence with Deep Learning. Cell.

[45] Rentzsch P., Schubach M., Shendure J., Kircher M. (2021). CADD-Splice—improving genome-wide variant effect prediction using deep learning-derived splice scores. Genome Med.

[46] Barash Y., Calarco J.A., Gao W., Pan Q., Wang X., Shai O. (2010). Deciphering the splicing code. Nature.

[47] Schärfen L., Neugebauer K.M. (2021). Transcription Regulation Through Nascent RNA Folding. J Mol Biol.

[48] Lai D., Proctor J.R., Meyer I.M. (2013). On the importance of cotranscriptional RNA structure formation. RNA.

[49] Kalinina M., Skvortsov D., Kalmykova S., Ivanov T., Dontsova O., Pervouchine D.D. (2021). Multiple competing RNA structures dynamically control alternative splicing in the human ATE1 gene. Nucleic Acids Res.

[50] Fong N., Kim H., Zhou Y., Ji X., Qiu J., Saldi T. (2014). Pre-mRNA splicing is facilitated by an optimal RNA polymerase II elongation rate. Genes Dev.

[51] Gerstberger S., Hafner M., Tuschl T. (2014). A census of human RNA-binding proteins. Nat Rev Genet.

[52] Hiller M., Zhang Z., Backofen R., Stamm S. (2007). Pre-mRNA secondary structures influence exon recognition. PLoS Genet.

[53] Maris C., Dominguez C., Allain F.-H.-T. (2005). The RNA recognition motif, a plastic RNA-binding platform to regulate post-transcriptional gene expression. FEBS J.

[54] Taliaferro J.M., Lambert N.J., Sudmant P.H., Dominguez D., Merkin J.J., Alexis M.S. (2016). RNA Sequence Context Effects Measured In Vitro Predict In Vivo Protein Binding and Regulation. Mol Cell.

[55] Li X., Quon G., Lipshitz H.D., Morris Q. (2010). Predicting in vivo binding sites of RNA-binding proteins using mRNA secondary structure. RNA.

[56] Jin Y., Yang Y., Zhang P. (2011). New insights into RNA secondary structure in the alternative splicing of pre-mRNAs. RNA Biol.

[57] Shepard P.J., Hertel K.J. (2008). Conserved RNA secondary structures promote alternative splicing. RNA.

[58] Buratti E., Baralle F.E. (2004). Influence of RNA secondary structure on the pre-mRNA splicing process. Mol Cell Biol.

[59] McManus C.J., Graveley B.R. (2011). RNA structure and the mechanisms of alternative splicing. Curr Opin Genet Dev.

[60] Shi H., Hoffman B.E., Lis J.T. (1997). A specific RNA hairpin loop structure binds the RNA recognition motifs of the Drosophila SR protein B52. Mol Cell Biol.

[61] Saha K., England W., Fernandez M.M., Biswas T., Spitale R.C., Ghosh G. (2020). Structural disruption of exonic stem-loops immediately upstream of the intron regulates mammalian splicing. Nucleic Acids Res.

[62] Hertel K.J. (2008). Combinatorial control of exon recognition. J Biol Chem.

[63] Warf M.B., Diegel J.V., von Hippel P.H., Berglund J.A. (2009). The protein factors MBNL1 and U2AF65 bind alternative RNA structures to regulate splicing. Proc Natl Acad Sci U S A.

[64] Warf M.B., Berglund J.A. (2007). MBNL binds similar RNA structures in the CUG repeats of myotonic dystrophy and its pre-mRNA substrate cardiac troponin T. RNA.

[65] Lee A.S.Y., Kranzusch P.J., Cate J.H.D. (2015). eIF3 targets cell-proliferation messenger RNAs for translational activation or repression. Nature.

[66] Benhalevy D., Gupta S.K., Danan C.H., Ghosal S., Sun H.-W., Kazemier H.G. (2017). The Human CCHC-type Zinc Finger Nucleic Acid-Binding Protein Binds G-Rich Elements in Target mRNA Coding Sequences and Promotes Translation. Cell Rep.

[67] Goering R., Hudish L.I., Guzman B.B., Raj N., Bassell G.J., Russ H.A. (2020). FMRP promotes RNA localization to neuronal projections through interactions between its RGG domain and G-quadruplex RNA sequences. Elife.

[68] Fukunaga T., Ozaki H., Terai G., Asai K., Iwasaki W., Kiryu H. (2014). CapR: revealing structural specificities of RNA-binding protein target recognition using CLIP-seq data. Genome Biol.

[69] Maticzka D., Lange S.J., Costa F., Backofen R. (2014). GraphProt: modeling binding preferences of RNA-binding proteins. Genome Biol.

[70] Lee D.S.M., Ghanem L.R., Barash Y. (2020). Integrative analysis reveals RNA G-quadruplexes in UTRs are selectively constrained and enriched for functional associations. Nat Commun.

[71] Marintchev A., Edmonds K.A., Marintcheva B., Hendrickson E., Oberer M., Suzuki C. (2009). Topology and regulation of the human eIF4A/4G/4H helicase complex in translation initiation. Cell.

[72] Pisareva V.P., Pisarev A.V., Komar A.A., Hellen C.U.T., Pestova T.V. (2008). Translation Initiation on Mammalian mRNAs with Structured 5′UTRs Requires DExH-Box Protein DHX29. Cell.

[73] Chakraborty P., Grosse F. (2011). Human DHX9 helicase preferentially unwinds RNA-containing displacement loops (R-loops) and G-quadruplexes. DNA Repair.

[74] Gulliver C, Hoffmann R, Baillie GS. The enigmatic helicase DHX9 and its association with the hallmarks of cancer. Future Sci OA 2020;7:FSO650. 10.2144/fsoa-2020-0140 PMC7787180 33437516

[75] Serikawa T., Spanos C., von Hacht A., Budisa N., Rappsilber J., Kurreck J. (2018). Comprehensive identification of proteins binding to RNA G-quadruplex motifs in the 5’ UTR of tumor-associated mRNAs. Biochimie.

[76] Fay M.M., Lyons S.M., Ivanov P. (2017). RNA G-Quadruplexes in Biology: Principles and Molecular Mechanisms. J Mol Biol.

[77] Chen M.C., Tippana R., Demeshkina N.A., Murat P., Balasubramanian S., Myong S. (2018). Structural basis of G-quadruplex unfolding by the DEAH/RHA helicase DHX36. Nature.

[78] Chen X., Yuan J., Xue G., Campanario S., Wang D., Wang W. (2021). Translational control by DHX36 binding to 5’UTR G-quadruplex is essential for muscle stem-cell regenerative functions. Nat Commun.

[79] Nie J., Jiang M., Zhang X., Tang H., Jin H., Huang X. (2015). Post-transcriptional Regulation of Nkx2-5 by RHAU in Heart Development. Cell Rep.

[80] Murat P., Marsico G., Herdy B., Ghanbarian A.T., Portella G., Balasubramanian S. (2018). RNA G-quadruplexes at upstream open reading frames cause DHX36- and DHX9-dependent translation of human mRNAs. Genome Biol.

[81] Ribeiro de Almeida C., Dhir S., Dhir A., Moghaddam A.E., Sattentau Q., Meinhart A. (2018). RNA Helicase DDX1 Converts RNA G-Quadruplex Structures into R-Loops to Promote IgH Class Switch Recombination. Mol Cell.

[82] Herdy B., Mayer C., Varshney D., Marsico G., Murat P., Taylor C. (2018). Analysis of NRAS RNA G-quadruplex binding proteins reveals DDX3X as a novel interactor of cellular G-quadruplex containing transcripts. Nucleic Acids Res.

[83] McRae E.K.S., Booy E.P., Moya-Torres A., Ezzati P., Stetefeld J., McKenna S.A. (2017). Human DDX21 binds and unwinds RNA guanine quadruplexes. Nucleic Acids Res.

[84] Dardenne E., Polay Espinoza M., Fattet L., Germann S., Lambert M.-P., Neil H. (2014). RNA helicases DDX5 and DDX17 dynamically orchestrate transcription, miRNA, and splicing programs in cell differentiation. Cell Rep.

[85] Fiorini F., Bagchi D., Le Hir H., Croquette V. (2015). Human Upf1 is a highly processive RNA helicase and translocase with RNP remodelling activities. Nat Commun.

[86] Kar A., Fushimi K., Zhou X., Ray P., Shi C., Chen X. (2011). RNA helicase p68 (DDX5) regulates tau exon 10 splicing by modulating a stem-loop structure at the 5’ splice site. Mol Cell Biol.

[87] Wu G., Xing Z., Tran E.J., Yang D. (2019). DDX5 helicase resolves G-quadruplex and is involved in gene transcriptional activation. Proc Natl Acad Sci U S A.

[88] Moindrot B., Cerase A., Coker H., Masui O., Grijzenhout A., Pintacuda G. (2015). A Pooled shRNA Screen Identifies Rbm15, Spen, and Wtap as Factors Required for Xist RNA-Mediated Silencing. Cell Rep.

[89] Van Nostrand E.L., Freese P., Pratt G.A., Wang X., Wei X., Xiao R. (2020). A large-scale binding and functional map of human RNA-binding proteins. Nature.

[90] Fei T., Chen Y., Xiao T., Li W., Cato L., Zhang P. (2017). Genome-wide CRISPR screen identifies HNRNPL as a prostate cancer dependency regulating RNA splicing. Proc Natl Acad Sci U S A.

[91] Wang E., Lu S.X., Pastore A., Chen X., Imig J., Chun-Wei Lee S. (2019). Targeting an RNA-Binding Protein Network in Acute Myeloid Leukemia. Cancer Cell.

[92] Bourgeois C.F., Mortreux F., Auboeuf D. (2016). The multiple functions of RNA helicases as drivers and regulators of gene expression. Nat Rev Mol Cell Biol.

[93] Caterino M., Paeschke K. (2021). Action and function of helicases on RNA G-quadruplexes. Methods.

[94] Mendoza O., Bourdoncle A., Boulé J.-B., Brosh R.M., Mergny J.-L. (2016). G-quadruplexes and helicases. Nucleic Acids Res.

[95] Paeschke K., Capra J.A., Zakian V.A. (2011). DNA replication through G-quadruplex motifs is promoted by the Saccharomyces cerevisiae Pif1 DNA helicase. Cell.

[96] Tran P.L.T., Pohl T.J., Chen C.-F., Chan A., Pott S., Zakian V.A. (2017). PIF1 family DNA helicases suppress R-loop mediated genome instability at tRNA genes. Nat Commun.

[97] Coin F., Oksenych V., Egly J.-M. (2007). Distinct roles for the XPB/p52 and XPD/p44 subcomplexes of TFIIH in damaged DNA opening during nucleotide excision repair. Mol Cell.

[98] Gray L.T., Vallur A.C., Eddy J., Maizels N. (2014). G quadruplexes are genomewide targets of transcriptional helicases XPB and XPD. Nat Chem Biol.

[99] Javadekar S.M., Nilavar N.M., Paranjape A., Das K., Raghavan S.C. (2020). Characterization of G-quadruplex antibody reveals differential specificity for G4 DNA forms. DNA Res.

[100] Nguyen G.H., Tang W., Robles A.I., Beyer R.P., Gray L.T., Welsh J.A. (2014). Regulation of gene expression by the BLM helicase correlates with the presence of G-quadruplex DNA motifs. Proc Natl Acad Sci U S A.

[101] Ellis N.A., Groden J., Ye T.Z., Straughen J., Lennon D.J., Ciocci S. (1995). The Bloom’s syndrome gene product is homologous to RecQ helicases. Cell.

[102] van Wietmarschen N., Merzouk S., Halsema N., Spierings D.C.J., Guryev V., Lansdorp P.M. (2018). BLM helicase suppresses recombination at G-quadruplex motifs in transcribed genes. Nat Commun.

[103] Marabitti V., Valenzisi P., Lillo G., Malacaria E., Palermo V., Pichierri P. (2022). R-Loop-Associated Genomic Instability and Implication of WRN and WRNIP1. Int J Mol Sci.

[104] Fry M., Loeb L.A. (1999). Human Werner Syndrome DNA Helicase Unwinds Tetrahelical Structures of the Fragile X Syndrome Repeat Sequence d(CGG)n*. J Biol Chem.

[105] Tang W., Robles A.I., Beyer R.P., Gray L.T., Nguyen G.H., Oshima J. (2016). The Werner syndrome RECQ helicase targets G4 DNA in human cells to modulate transcription. Hum Mol Genet.

[106] Fidaleo M., Svetoni F., Volpe E., Miñana B., Caporossi D., Paronetto M.P. (2015). Genotoxic stress inhibits Ewing sarcoma cell growth by modulating alternative pre-mRNA processing of the RNA helicase DHX9. Oncotarget.

[107] Cristini A., Groh M., Kristiansen M.S., Gromak N. (2018). RNA/DNA Hybrid Interactome Identifies DXH9 as a Molecular Player in Transcriptional Termination and R-Loop-Associated DNA Damage. Cell Rep.

[108] Jain A., Bacolla A., Chakraborty P., Grosse F., Vasquez K.M. (2010). Human DHX9 helicase unwinds triple-helical DNA structures. Biochemistry.

[109] Jain A., Bacolla A., Del Mundo I.M., Zhao J., Wang G., Vasquez K.M. (2013). DHX9 helicase is involved in preventing genomic instability induced by alternatively structured DNA in human cells. Nucleic Acids Res.

[110] Aktaş T., Avşar Ilık İ., Maticzka D., Bhardwaj V., Pessoa Rodrigues C., Mittler G. (2017). DHX9 suppresses RNA processing defects originating from the Alu invasion of the human genome. Nature.

[111] Iwamoto F., Stadler M., Chalupníková K., Oakeley E., Nagamine Y. (2008). Transcription-dependent nucleolar cap localization and possible nuclear function of DExH RNA helicase RHAU. Exp Cell Res.

[112] Huang W., Smaldino P.J., Zhang Q., Miller L.D., Cao P., Stadelman K. (2011). Yin Yang 1 contains G-quadruplex structures in its promoter and 5′-UTR and its expression is modulated by G4 resolvase 1. Nucleic Acids Res.

[113] Sauer M., Juranek S.A., Marks J., De Magis A., Kazemier H.G., Hilbig D. (2019). DHX36 prevents the accumulation of translationally inactive mRNAs with G4-structures in untranslated regions. Nat Commun.

[114] Liu G., Du W., Xu H., Sun Q., Tang D., Zou S. (2020). RNA G-quadruplex regulates microRNA-26a biogenesis and function. J Hepatol.

[115] Booy E.P., Meier M., Okun N., Novakowski S.K., Xiong S., Stetefeld J. (2012). The RNA helicase RHAU (DHX36) unwinds a G4-quadruplex in human telomerase RNA and promotes the formation of the P1 helix template boundary. Nucleic Acids Res.

[116] Decorsière A., Cayrel A., Vagner S., Millevoi S. (2011). Essential role for the interaction between hnRNP H/F and a G quadruplex in maintaining p53 pre-mRNA 3’-end processing and function during DNA damage. Genes Dev.

[117] Herviou P., Le Bras M., Dumas L., Hieblot C., Gilhodes J., Cioci G. (2020). hnRNP H/F drive RNA G-quadruplex-mediated translation linked to genomic instability and therapy resistance in glioblastoma. Nat Commun.

[118] Fukuda T., Yamagata K., Fujiyama S., Matsumoto T., Koshida I., Yoshimura K. (2007). DEAD-box RNA helicase subunits of the Drosha complex are required for processing of rRNA and a subset of microRNAs. Nat Cell Biol.

[119] Lambert M.-P., Terrone S., Giraud G., Benoit-Pilven C., Cluet D., Combaret V. (2018). The RNA helicase DDX17 controls the transcriptional activity of REST and the expression of proneural microRNAs in neuronal differentiation. Nucleic Acids Res.

[120] Kim D.-S., Camacho C.V., Nagari A., Malladi V.S., Challa S., Kraus W.L. (2019). Activation of PARP-1 by snoRNAs Controls Ribosome Biogenesis and Cell Growth via the RNA Helicase DDX21. Mol Cell.

[121] Zhang Z., Kim T., Bao M., Facchinetti V., Jung S.Y., Ghaffari A.A. (2011). DDX1, DDX21, and DHX36 helicases form a complex with the adaptor molecule TRIF to sense dsRNA in dendritic cells. Immunity.

[122] Dong Y., Ye W., Yang J., Han P., Wang Y., Ye C. (2016). DDX21 translocates from nucleus to cytoplasm and stimulates the innate immune response due to dengue virus infection. Biochem Biophys Res Commun.

[123] Sénéchal P., Robert F., Cencic R., Yanagiya A., Chu J., Sonenberg N. (2021). Assessing eukaryotic initiation factor 4F subunit essentiality by CRISPR-induced gene ablation in the mouse. Cell Mol Life Sci.

[124] Mosler T., Conte F., Longo G.M.C., Mikicic I., Kreim N., Möckel M.M. (2021). R-loop proximity proteomics identifies a role of DDX41 in transcription-associated genomic instability. Nat Commun.

[125] Pérez-Calero C., Bayona-Feliu A., Xue X., Barroso S.I., Muñoz S., González-Basallote V.M. (2020). UAP56/DDX39B is a major cotranscriptional RNA-DNA helicase that unwinds harmful R loops genome-wide. Genes Dev.

[126] Skourti-Stathaki K., Proudfoot N.J. (2014). A double-edged sword: R loops as threats to genome integrity and powerful regulators of gene expression. Genes Dev.

[127] Skourti-Stathaki K., Proudfoot N.J., Gromak N. (2011). Human senataxin resolves RNA/DNA hybrids formed at transcriptional pause sites to promote Xrn2-dependent termination. Mol Cell.

[128] Hirose T., Ideue T., Nagai M., Hagiwara M., Shu M.-D., Steitz J.A. (2006). A spliceosomal intron binding protein, IBP160, links position-dependent assembly of intron-encoded box C/D snoRNP to pre-mRNA splicing. Mol Cell.

[129] Sollier J., Stork C.T., García-Rubio M.L., Paulsen R.D., Aguilera A., Cimprich K.A. (2014). Transcription-coupled nucleotide excision repair factors promote R-loop-induced genome instability. Mol Cell.

[130] Akay A., Di Domenico T., Suen K.M., Nabih A., Parada G.E., Larance M. (2017). The Helicase Aquarius/EMB-4 Is Required to Overcome Intronic Barriers to Allow Nuclear RNAi Pathways to Heritably Silence Transcription. Dev Cell.

[131] Balasubramanian S., Hurley L.H., Neidle S. (2011). Targeting G-quadruplexes in gene promoters: a novel anticancer strategy?. Nat Rev Drug Discov.

[132] Georgakopoulos-Soares I, Parada GE, Wong HY, Miska EA, Kwok CK, Hemberg M. Alternative splicing modulation by G-quadruplexes n.d. https://doi.org/10.1101/700575. 10.1038/s41467-022-30071-7 PMC9065059 35504902

[133] Gomez D., Lemarteleur T., Lacroix L., liet P., Mergny J-L., Riou J-F. (2004). Telomerase downregulation induced by the G-quadruplex ligand 12459 in A549 cells is mediated by hTERT RNA alternative splicing. Nucleic Acids Res.

[134] Didiot M.-C., Tian Z., Schaeffer C., Subramanian M., Mandel J.-L., Moine H. (2008). The G-quartet containing FMRP binding site in FMR1 mRNA is a potent exonic splicing enhancer. Nucleic Acids Res.

[135] Marcel V., Tran P.L.T., Sagne C., Martel-Planche G., Vaslin L., Teulade-Fichou M.-P. (2011). G-quadruplex structures in TP53 intron 3: role in alternative splicing and in production of p53 mRNA isoforms. Carcinogenesis.

[136] Ribeiro M.M., Teixeira G.S., Martins L., Marques M.R., de Souza A.P., Line S.R.P. (2015). G-quadruplex formation enhances splicing efficiency of PAX9 intron 1. Hum Genet.

[137] Huang H., Zhang J., Harvey S.E., Hu X., Cheng C. (2017). RNA G-quadruplex secondary structure promotes alternative splicing via the RNA-binding protein hnRNPF. Genes Dev.

[138] Bartys N, Kierzek R, Lisowiec-Wachnicka J. The regulation properties of RNA secondary structure in alternative splicing. Biochim Biophys Acta Gene Regul Mech 2019:194401. 10.1016/j.bbagrm.2019.07.002 31323437

[139] Izumi H., Funa K. (2019). Telomere Function and the G-Quadruplex Formation are Regulated by hnRNP U. Cells.

[140] Zhang J., Harvey S.E., Cheng C. (2019). A high-throughput screen identifies small molecule modulators of alternative splicing by targeting RNA G-quadruplexes. Nucleic Acids Res.

[141] Kalmykova S., Kalinina M., Denisov S., Mironov A., Skvortsov D., Guigó R. (2021). Conserved long-range base pairings are associated with pre-mRNA processing of human genes. Nat Commun.

[142] Pervouchine D.D., Khrameeva E.E., Pichugina M.Y., Nikolaienko O.V., Gelfand M.S., Rubtsov P.M. (2012). Evidence for widespread association of mammalian splicing and conserved long-range RNA structures. RNA.

[143] Graveley B.R. (2005). Mutually exclusive splicing of the insect Dscam pre-mRNA directed by competing intronic RNA secondary structures. Cell.

[144] Yang Y., Zhan L., Zhang W., Sun F., Wang W., Tian N. (2011). RNA secondary structure in mutually exclusive splicing. Nat Struct Mol Biol.

[145] Miriami E., Margalit H., Sperling R. (2003). Conserved sequence elements associated with exon skipping. Nucleic Acids Res.

[146] Howe K.J., Ares M. (1997). Intron self-complementarity enforces exon inclusion in a yeast pre-mRNA. Proc Natl Acad Sci U S A.

[147] Lev-Maor G., Ram O., Kim E., Sela N., Goren A., Levanon E.Y. (2008). Intronic Alus influence alternative splicing. PLoS Genet.

[148] Jeck W.R., Sorrentino J.A., Wang K., Slevin M.K., Burd C.E., Liu J. (2013). Circular RNAs are abundant, conserved, and associated with ALU repeats. RNA.

[149] Lin C.-L., Taggart A.J., Lim K.H., Cygan K.J., Ferraris L., Creton R. (2016). RNA structure replaces the need for U2AF2 in splicing. Genome Res.

[150] Lewis C.J.T., Pan T., Kalsotra A. (2017). RNA modifications and structures cooperate to guide RNA-protein interactions. Nat Rev Mol Cell Biol.

[151] Lovci M.T., Ghanem D., Marr H., Arnold J., Gee S., Parra M. (2013). Rbfox proteins regulate alternative mRNA splicing through evolutionarily conserved RNA bridges. Nat Struct Mol Biol.

[152] Muh S.J., Hovhannisyan R.H., Carstens R.P. (2002). A Non-sequence-specific double-stranded RNA structural element regulates splicing of two mutually exclusive exons of fibroblast growth factor receptor 2 (FGFR2). J Biol Chem.

[153] Muro A.F., Caputi M., Pariyarath R., Pagani F., Buratti E., Baralle F.E. (1999). Regulation of fibronectin EDA exon alternative splicing: possible role of RNA secondary structure for enhancer display. Mol Cell Biol.

[154] Buratti E., Muro A.F., Giombi M., Gherbassi D., Iaconcig A., Baralle F.E. (2004). RNA folding affects the recruitment of SR proteins by mouse and human polypurinic enhancer elements in the fibronectin EDA exon. Mol Cell Biol.

[155] Libri D., Balvay L., Fiszman M.Y. (1992). In vivo splicing of the beta tropomyosin pre-mRNA: a role for branch point and donor site competition. Mol Cell Biol.

[156] Higashide S., Morikawa K., Okumura M., Kondo S., Ogata M., Murakami T. (2004). Identification of regulatory cis-acting elements for alternative splicing of presenilin 2 exon 5 under hypoxic stress conditions. J Neurochem.

[157] Meyer M., Plass M., Pérez-Valle J., Eyras E., Vilardell J. (2011). Deciphering 3′ss Selection in the Yeast Genome Reveals an RNA Thermosensor that Mediates Alternative Splicing. Mol Cell.

[158] Wu X., Bartel D.P. (2017). Widespread Influence of 3′-End Structures on Mammalian mRNA Processing and Stability. Cell.

[159] Mauger D.M., Joseph Cabral B., Presnyak V., Su S.V., Reid D.W., Goodman B. (2019). mRNA structure regulates protein expression through changes in functional half-life. Proc Natl Acad Sci.

[160] Hia F., Yang S.F., Shichino Y., Yoshinaga M., Murakawa Y., Vandenbon A. (2019). Codon bias confers stability to human mRNAs. EMBO Rep.

[161] Agarwal V., Subtelny A.O., Thiru P., Ulitsky I., Bartel D.P. (2018). Predicting microRNA targeting efficacy in Drosophila. Genome Biol.

[162] Courel M., Clément Y., Bossevain C., Foretek D., Vidal Cruchez O., Yi Z. (2019). GC content shapes mRNA storage and decay in human cells. Elife.

[163] Geisberg J.V., Moqtaderi Z., Fan X., Ozsolak F., Struhl K. (2014). Global analysis of mRNA isoform half-lives reveals stabilizing and destabilizing elements in yeast. Cell.

[164] Carrier T.A., Keasling J.D. (1997). Engineering mRNA stability in E. coli by the addition of synthetic hairpins using a 5’ cassette system. Biotechnol Bioeng.

[165] Siegel DA, Le Tonqueze O, Biton A, Zaitlen N, Erle DJ. Massively Parallel Analysis of Human 3′ UTRs Reveals that AU-Rich Element Length and Registration Predict mRNA Destabilization n.d. https://doi.org/10.1101/2020.02.12.945063. 10.1093/g3journal/jkab404 PMC8728028 34849835

[166] Kertesz M., Iovino N., Unnerstall U., Gaul U., Segal E. (2007). The role of site accessibility in microRNA target recognition. Nat Genet.

[167] Carrier T.A., Keasling J.D. (1997). Controlling messenger RNA stability in bacteria: strategies for engineering gene expression. Biotechnol Prog.

[168] Carrier T., Jones K.L., Keasling J.D. (1998). mRNA stability and plasmid copy number effects on gene expression from an inducible promoter system. Biotechnol Bioeng.

[169] Leppek K., Schott J., Reitter S., Poetz F., Hammond M.C., Stoecklin G. (2013). Roquin Promotes Constitutive mRNA Decay via a Conserved Class of Stem-Loop Recognition Motifs. Cell.

[170] Codutti L., Leppek K., Zálešák J., Windeisen V., Masiewicz P., Stoecklin G. (2015). A Distinct, Sequence-Induced Conformation Is Required for Recognition of the Constitutive Decay Element RNA by Roquin. Structure.

[171] Inoue F., Ahituv N. (2015). Decoding enhancers using massively parallel reporter assays. Genomics.

[172] Gordon M.G., Inoue F., Martin B., Schubach M., Agarwal V., Whalen S. (2020). lentiMPRA and MPRAflow for high-throughput functional characterization of gene regulatory elements. Nat Protoc.

[173] Georgakopoulos-Soares I., Jain N., Gray J.M., Hemberg M. (2017). MPRAnator: a web-based tool for the design of massively parallel reporter assay experiments. Bioinformatics.

[174] Ashuach T., Fischer D.S., Kreimer A., Ahituv N., Theis F.J., Yosef N. (2019). MPRAnalyze: statistical framework for massively parallel reporter assays. Genome Biol.

[175] Hershey J.W.B., Sonenberg N., Mathews M.B. (2012). Principles of translational control: an overview. Cold Spring Harb Perspect Biol.

[176] Ingolia N.T., Hussmann J.A., Weissman J.S. (2019). Ribosome Profiling: Global Views of Translation. Cold Spring Harb Perspect Biol.

[177] Sonenberg N., Hinnebusch A.G. (2009). Regulation of translation initiation in eukaryotes: mechanisms and biological targets. Cell.

[178] Silvera D., Formenti S.C., Schneider R.J. (2010). Translational control in cancer. Nat Rev Cancer.

[179] Shirokikh NE, Preiss T. Translation initiation by cap‐dependent ribosome recruitment: Recent insights and open questions. WIREs RNA 2018;9. https://doi.org/10.1002/wrna.1473. 10.1002/wrna.1473 29624880

[180] Gebauer F., Hentze M.W. (2004). Molecular mechanisms of translational control. Nat Rev Mol Cell Biol.

[181] Hinnebusch A.G. (2014). The Scanning Mechanism of Eukaryotic Translation Initiation. Annu Rev Biochem.

[182] Kwan T., Thompson S.R. (2019). Noncanonical Translation Initiation in Eukaryotes. Cold Spring Harbor Perspect Biol.

[183] Lozano G., Martínez-Salas E. (2015). Structural insights into viral IRES-dependent translation mechanisms. Curr Opin Virol.

[184] Dvir S., Velten L., Sharon E., Zeevi D., Carey L.B., Weinberger A. (2013). Deciphering the rules by which 5’-UTR sequences affect protein expression in yeast. Proc Natl Acad Sci U S A.

[185] Lamping E., Niimi M., Cannon R.D. (2013). Small, synthetic, GC-rich mRNA stem-loop modules 5′ proximal to the AUG start-codon predictably tune gene expression in yeast. Microb Cell Fact.

[186] Pelletier J., Sonenberg N. (1985). Insertion mutagenesis to increase secondary structure within the 5’ noncoding region of a eukaryotic mRNA reduces translational efficiency. Cell.

[187] Beaudoin J.-D., Novoa E.M., Vejnar C.E., Yartseva V., Takacs C.M., Kellis M. (2018). Analyses of mRNA structure dynamics identify embryonic gene regulatory programs. Nat Struct Mol Biol.

[188] Shah P., Ding Y., Niemczyk M., Kudla G., Plotkin J.B. (2013). Rate-limiting steps in yeast protein translation. Cell.

[189] Yu C.-H., Dang Y., Zhou Z., Wu C., Zhao F., Sachs M.S. (2015). Codon Usage Influences the Local Rate of Translation Elongation to Regulate Co-translational Protein Folding. Mol Cell.

[190] Kozak M. (2005). Regulation of translation via mRNA structure in prokaryotes and eukaryotes. Gene.

[191] Gu W., Zhou T., Wilke C.O. (2010). A universal trend of reduced mRNA stability near the translation-initiation site in prokaryotes and eukaryotes. PLoS Comput Biol.

[192] Araujo P.R., Yoon K., Ko D., Smith A.D., Qiao M., Suresh U. (2012). Before It Gets Started: Regulating Translation at the 5′ UTR. Comp Funct Genomics.

[193] Zu T., Gibbens B., Doty N.S., Gomes-Pereira M., Huguet A., Stone M.D. (2011). Non-ATG–initiated translation directed by microsatellite expansions. Proc Natl Acad Sci.

[194] Rogozin I.B., Kochetov A.V., Kondrashov F.A., Koonin E.V., Milanesi L. (2001). Presence of ATG triplets in 5’ untranslated regions of eukaryotic cDNAs correlates with a “weak” context of the start codon. Bioinformatics.

[195] Kochetov A.V., Sarai A., Rogozin I.B., Shumny V.K., Kolchanov N.A. (2005). The role of alternative translation start sites in the generation of human protein diversity. Mol Genet Genomics.

[196] Dominguez D., Freese P., Alexis M.S., Su A., Hochman M., Palden T. (2018). Sequence, Structure, and Context Preferences of Human RNA Binding Proteins. Mol Cell.

[197] Kozak M. (1989). Circumstances and mechanisms of inhibition of translation by secondary structure in eucaryotic mRNAs. Mol Cell Biol.

[198] Honda M., Brown E.A., Lemon S.M. (1996). Stability of a stem-loop involving the initiator AUG controls the efficiency of internal initiation of translation on hepatitis C virus RNA. RNA.

[199] Fernández N., Fernandez-Miragall O., Ramajo J., García-Sacristán A., Bellora N., Eyras E. (2011). Structural basis for the biological relevance of the invariant apical stem in IRES-mediated translation. Nucleic Acids Res.

[200] Komar A.A., Hatzoglou M. (2011). Cellular IRES-mediated translation. Cell Cycle.

[201] Serganov A., Nudler E. (2013). A Decade of Riboswitches Cell.

[202] Winkler W., Nahvi A., Breaker R.R. (2002). Thiamine derivatives bind messenger RNAs directly to regulate bacterial gene expression. Nature.

[203] Caron M.-P., Bastet L., Lussier A., Simoneau-Roy M., Massé E., Lafontaine D.A. (2012). Dual-acting riboswitch control of translation initiation and mRNA decay. Proc Natl Acad Sci U S A.

[204] Hollands K., Proshkin S., Sklyarova S., Epshtein V., Mironov A., Nudler E. (2012). Riboswitch control of Rho-dependent transcription termination. Proc Natl Acad Sci U S A.

[205] Li S., Breaker R.R. (2013). Eukaryotic TPP riboswitch regulation of alternative splicing involving long-distance base pairing. Nucleic Acids Res.

[206] Cheah M.T., Wachter A., Sudarsan N., Breaker R.R. (2007). Control of alternative RNA splicing and gene expression by eukaryotic riboswitches. Nature.

[207] Babendure J.R., Babendure J.L., Ding J.-H., Tsien R.Y. (2006). Control of mammalian translation by mRNA structure near caps. RNA.

[208] Sobczak K., Krzyzosiak W.J. (2002). Structural determinants of BRCA1 translational regulation. J Biol Chem.

[209] Buccitelli C., Selbach M. (2020). mRNAs, proteins and the emerging principles of gene expression control. Nat Rev Genet.

[210] Kozak M. (1986). Influences of mRNA secondary structure on initiation by eukaryotic ribosomes. Proc Natl Acad Sci U S A.

[211] Kozak M. (1990). Downstream secondary structure facilitates recognition of initiator codons by eukaryotic ribosomes. Proc Natl Acad Sci U S A.

[212] Kozak M. (1989). Context effects and inefficient initiation at non-AUG codons in eucaryotic cell-free translation systems. Mol Cell Biol.

[213] Vega Laso M.R., Zhu D., Sagliocco F., Brown A.J., Tuite M.F., McCarthy J.E. (1993). Inhibition of translational initiation in the yeast Saccharomyces cerevisiae as a function of the stability and position of hairpin structures in the mRNA leader. J Biol Chem.

[214] Thomson A.M., Rogers J.T., Leedman P.J. (1999). Iron-regulatory proteins, iron-responsive elements and ferritin mRNA translation. Int J Biochem Cell Biol.

[215] Walker M.J., Shortridge M.D., Albin D.D., Cominsky L.Y., Varani G. (2020). Structure of the RNA Specialized Translation Initiation Element that Recruits eIF3 to the 5′-UTR of c-Jun. J Mol Biol.

[216] Cuperus J.T., Groves B., Kuchina A., Rosenberg A.B., Jojic N., Fields S. (2017). Deep learning of the regulatory grammar of yeast 5’ untranslated regions from 500,000 random sequences. Genome Res.

[217] Huppert J.L., Bugaut A., Kumari S., Balasubramanian S. (2008). G-quadruplexes: the beginning and end of UTRs. Nucleic Acids Res.

[218] Halder K., Wieland M., Hartig J.S. (2009). Predictable suppression of gene expression by 5’-UTR-based RNA quadruplexes. Nucleic Acids Res.

[219] Kumari S., Bugaut A., Huppert J.L., Balasubramanian S. (2007). An RNA G-quadruplex in the 5’ UTR of the NRAS proto-oncogene modulates translation. Nat Chem Biol.

[220] Blice-Baum A.C., Mihailescu M.-R. (2014). Biophysical characterization of G-quadruplex forming FMR1 mRNA and of its interactions with different fragile X mental retardation protein isoforms. RNA.

[221] Morris M.J., Basu S. (2009). An unusually stable G-quadruplex within the 5’-UTR of the MT3 matrix metalloproteinase mRNA represses translation in eukaryotic cells. Biochemistry.

[222] Arora A., Dutkiewicz M., Scaria V., Hariharan M., Maiti S., Kurreck J. (2008). Inhibition of translation in living eukaryotic cells by an RNA G-quadruplex motif. RNA.

[223] Shahid R., Bugaut A., Balasubramanian S. (2010). The BCL-2 5’ untranslated region contains an RNA G-quadruplex-forming motif that modulates protein expression. Biochemistry.

[224] Agarwal T., Jayaraj G., Pandey S.P., Agarwala P., Maiti S. (2012). RNA G-quadruplexes: G-quadruplexes with “U” turns. Curr Pharm Des.

[225] Bonnal S., Schaeffer C., Créancier L., Clamens S., Moine H., Prats A.-C. (2003). A Single Internal Ribosome Entry Site Containing a G Quartet RNA Structure Drives Fibroblast Growth Factor 2 Gene Expression at Four Alternative Translation Initiation Codons. J Biol Chem.

[226] Koukouraki P., Doxakis E. (2016). Constitutive translation of human α-synuclein is mediated by the 5’-untranslated region. Open Biol.

[227] Morris M.J., Negishi Y., Pazsint C., Schonhoft J.D., Basu S. (2010). An RNA G-quadruplex is essential for cap-independent translation initiation in human VEGF IRES. J Am Chem Soc.

[228] Schaeffer C., Bardoni B., Mandel J.L., Ehresmann B., Ehresmann C., Moine H. (2001). The fragile X mental retardation protein binds specifically to its mRNA via a purine quartet motif. EMBO J.

[229] Bhattacharyya D., Diamond P., Basu S. (2015). An Independently folding RNA G-quadruplex domain directly recruits the 40S ribosomal subunit. Biochemistry.

[230] Niu K., Zhang X., Song Q., Feng Q. (2022). G-Quadruplex Regulation of mRNA Translation by RBM4. Int J Mol Sci.

[231] Cammas A., Dubrac A., Morel B., Lamaa A., Touriol C., Teulade-Fichou M.-P. (2015). Stabilization of the G-quadruplex at the VEGF IRES represses cap-independent translation. RNA Biol.

[232] Hu X.-X., Wang S.-Q., Gan S.-Q., Liu L., Zhong M.-Q., Jia M.-H. (2021). A Small Ligand That Selectively Binds to the G-quadruplex at the Human Vascular Endothelial Growth Factor Internal Ribosomal Entry Site and Represses the Translation. Front Chem.

[233] Jia L., Mao Y., Ji Q., Dersh D., Yewdell J.W., Qian S.-B. (2020). Decoding mRNA translatability and stability from the 5’ UTR. Nat Struct Mol Biol.

[234] Jackson R.J., Hellen C.U.T., Pestova T.V. (2010). The mechanism of eukaryotic translation initiation and principles of its regulation. Nat Rev Mol Cell Biol.

[235] Georgakopoulos-Soares I., Morganella S., Jain N., Hemberg M., Nik-Zainal S. (2018). Noncanonical secondary structures arising from non-B DNA motifs are determinants of mutagenesis. Genome Res.

[236] Georgakopoulos-Soares I., Victorino J., Parada G.E., Agarwal V., Zhao J., Wong H.Y. (2022). High-throughput characterization of the role of non-B DNA motifs on promoter function. Cell Genomics.

[237] Gan W., Guan Z., Liu J., Gui T., Shen K., Manley J.L. (2011). R-loop-mediated genomic instability is caused by impairment of replication fork progression. Genes Dev.

[238] García-Muse T., Aguilera A. (2019). R Loops: From Physiological to Pathological Roles. Cell.

[239] Bonnet A., Grosso A.R., Elkaoutari A., Coleno E., Presle A., Sridhara S.C. (2017). Introns Protect Eukaryotic Genomes from Transcription-Associated Genetic Instability. Mol Cell.

[240] Petermann E., Lan L., Zou L. (2022). Sources, resolution and physiological relevance of R-loops and RNA–DNA hybrids. Nat Rev Mol Cell Biol.

[241] Li X., Manley J.L. (2005). Inactivation of the SR protein splicing factor ASF/SF2 results in genomic instability. Cell.

[242] Nguyen H.D., Zou L., Graubert T.A. (2019). Targeting R-loop-associated ATR response in myelodysplastic syndrome. Oncotarget.

[243] Nguyen H.D., Leong W.Y., Li W., Reddy P.N.G., Sullivan J.D., Walter M.J. (2018). Spliceosome Mutations Induce R Loop-Associated Sensitivity to ATR Inhibition in Myelodysplastic Syndromes. Cancer Res.

[244] Liu F., Gong C.-X. (2008). Tau exon 10 alternative splicing and tauopathies. Mol Neurodegener.

[245] Donahue C.P., Muratore C., Wu J.Y., Kosik K.S., Wolfe M.S. (2006). Stabilization of the tau exon 10 stem loop alters pre-mRNA splicing. J Biol Chem.

[246] Rogers J.T., Randall J.D., Cahill C.M., Eder P.S., Huang X., Gunshin H. (2002). An iron-responsive element type II in the 5’-untranslated region of the Alzheimer's amyloid precursor protein transcript. J Biol Chem.

[247] Friedlich A.L., Tanzi R.E., Rogers J.T. (2007). The 5’-untranslated region of Parkinson's disease alpha-synuclein messengerRNA contains a predicted iron responsive element. Mol Psychiatry.

[248] Garcia-Lopez A., Tessaro F., Jonker H.R.A., Wacker A., Richter C., Comte A. (2018). Targeting RNA structure in SMN2 reverses spinal muscular atrophy molecular phenotypes. Nat Commun.

[249] Sulovari A., Li R., Audano P.A., Porubsky D., Vollger M.R., Logsdon G.A. (2019). Human-specific tandem repeat expansion and differential gene expression during primate evolution. Proc Natl Acad Sci U S A.

[250] Georgakopoulos-Soares I., Chartoumpekis D.V., Kyriazopoulou V., Zaravinos A. (2020). EMT Factors and Metabolic Pathways in Cancer. Front Oncol.

[251] Svitkin Y.V., Pause A., Haghighat A., Pyronnet S., Witherell G., Belsham G.J. (2001). The requirement for eukaryotic initiation factor 4A (elF4A) in translation is in direct proportion to the degree of mRNA 5’ secondary structure. RNA.

[252] Wolfe A.L., Singh K., Zhong Y., Drewe P., Rajasekhar V.K., Sanghvi V.R. (2014). RNA G-quadruplexes cause eIF4A-dependent oncogene translation in cancer. Nature.

[253] Green K.M., Glineburg M.R., Kearse M.G., Flores B.N., Linsalata A.E., Fedak S.J. (2017). RAN translation at C9orf72-associated repeat expansions is selectively enhanced by the integrated stress response. Nat Commun.

[254] Haeusler A.R., Donnelly C.J., Periz G., Simko E.A.J., Shaw P.G., Kim M.-S. (2014). C9orf72 nucleotide repeat structures initiate molecular cascades of disease. Nature.

[255] Reddy K., Zamiri B., Stanley S.Y.R., Macgregor R.B., Pearson C.E. (2013). The disease-associated r(GGGGCC)n repeat from the C9orf72 gene forms tract length-dependent uni- and multimolecular RNA G-quadruplex structures. J Biol Chem.

[256] Fratta P., Mizielinska S., Nicoll A.J., Zloh M., Fisher E.M.C., Parkinson G. (2012). C9orf72 hexanucleotide repeat associated with amyotrophic lateral sclerosis and frontotemporal dementia forms RNA G-quadruplexes. Sci Rep.

[257] Gitler A.D., Tsuiji H. (2016). There has been an awakening: Emerging mechanisms of C9orf72 mutations in FTD/ALS. Brain Res.

[258] Liu H., Lu Y.-N., Paul T., Periz G., Banco M.T., Ferré-D’Amaré A.R. (2021). A Helicase Unwinds Hexanucleotide Repeat RNA G-Quadruplexes and Facilitates Repeat-Associated Non-AUG Translation. J Am Chem Soc.

[259] Ishiguro A., Kimura N., Noma T., Shimo-Kon R., Ishihama A., Kon T. (2020). Molecular dissection of ALS-linked TDP-43 – Involvement of the Gly-rich domain in interaction with G-quadruplex mRNA. FEBS Lett.

[260] Liu X., Xu Y. (2018). HnRNPA1 Specifically Recognizes the Base of Nucleotide at the Loop of RNA G-Quadruplex. Molecules.

[261] Scalabrin M., Frasson I., Ruggiero E., Perrone R., Tosoni E., Lago S. (2017). The cellular protein hnRNP A2/B1 enhances HIV-1 transcription by unfolding LTR promoter G-quadruplexes. Sci Rep.

[262] Mori K., Lammich S., Mackenzie I.R.A., Forné I., Zilow S., Kretzschmar H. (2013). hnRNP A3 binds to GGGGCC repeats and is a constituent of p62-positive/TDP43-negative inclusions in the hippocampus of patients with C9orf72 mutations. Acta Neuropathol.

[263] Takahama K., Kino K., Arai S., Kurokawa R., Oyoshi T. (2011). Identification of Ewing’s sarcoma protein as a G-quadruplex DNA- and RNA-binding protein. FEBS J.

[264] Zamiri B., Reddy K., Macgregor R.B., Pearson C.E. (2014). TMPyP4 porphyrin distorts RNA G-quadruplex structures of the disease-associated r(GGGGCC)n repeat of the C9orf72 gene and blocks interaction of RNA-binding proteins. J Biol Chem.

[265] Simone R., Balendra R., Moens T.G., Preza E., Wilson K.M., Heslegrave A. (2018). G-quadruplex-binding small molecules ameliorate FTD/ALS pathology and. EMBO Mol Med.

[266] Masters C.L., Bateman R., Blennow K., Rowe C.C., Sperling R.A., Cummings J.L. (2015). Alzheimer’s disease. Nat Rev Dis Primers.

[267] Fisette J.-F., Montagna D.R., Mihailescu M.-R., Wolfe M.S. (2012). A G-rich element forms a G-quadruplex and regulates BACE1 mRNA alternative splicing. J Neurochem.

[268] Lammich S., Kamp F., Wagner J., Nuscher B., Zilow S., Ludwig A.-K. (2011). Translational repression of the disintegrin and metalloprotease ADAM10 by a stable G-quadruplex secondary structure in its 5’-untranslated region. J Biol Chem.

